# ﻿Midget cave spiders (Araneae, Leptonetidae) from Jiangxi and Fujian Province, China

**DOI:** 10.3897/zookeys.1189.111041

**Published:** 2024-01-18

**Authors:** Bin-Lu Liu, Yan-bin Yao, Zi-Min Jiang, Yong-hong Xiao, Ke-Ke Liu

**Affiliations:** 1 College of Life Science, Jinggangshan University, Ji'an 343009, Jiangxi, China Jinggangshan University Ji'an China; 2 Jinshan College of Fujian Agriculture and Forestry University, Fuzhou 350007, Fujian, China Jinshan College of Fujian Agriculture and Forestry University Fuzhou China

**Keywords:** Asia, biodiversity, distribution, leptonetid spiders, new species, taxonom

## Abstract

Eleven leptonetid species belonging to four genera collected in Jiangxi and Fujian Provinces, China are presented. Ten new species of midget cave spiders from southern China are diagnosed, described, and illustrated: *Leptoneteladawu* Yao & Liu, **sp. nov.**, *L.yuanhaoi* Yao & Liu, **sp. nov.** and *L.zuojiashanensis* Yao & Liu, **sp. nov.** from Jiangxi; *Longileptonetaguadunensis* Yao & Liu, **sp. nov.**, *L.huboliao* Yao & Liu, **sp. nov.**, *L.jiaxiani* Yao & Liu, **sp. nov.**, *L.letuensis* Yao & Liu, **sp. nov.**, *L.renzhouensis* Yao & Liu, **sp. nov.**, *L.tianmenensis* Yao & Liu, **sp. nov.**, and *Pararanamingxuani* Yao & Liu, **sp. nov.** from Fujian. Furthermore, *Falcileptonetamonodactyla* (Yin, Wang & Wang, 1984) is recorded from Jiangxi province for the first time. Distributions records are given for all investigated species.

## ﻿Introduction

The midget cave spider family Leptonetidae Simon, 1890 is one of the smallest taxa in haplogyne spider families, with 374 species belonging to 22 genera (WSC 2023). At present, eight genera and 135 species are known from China. Among these genera, species of *Leptonetela* Kratochvíl, 1978 have been reported as being the most diverse in China.

Most records and descriptions of this family from China were contributed by the Chinese arachnologist Shu-Qiang Li and his team, such as of the genera *Jingneta* Wang & Li, 2020, *Leptonetela*, *Longileptoneta* Seo, 2015, *Pararana* Lin & Li, 2022 and *Rhyssoleptoneta* Tong & Li, 2007 (Wang and Li 2011; Wang et al. 2017, 2020; Lan et al. 2021; Zhu and Li 2021; Lin et al. 2022). In addition, many more genera have been recorded or described from China by other authors with eight species from northern provinces (Tong and Li 2008; Wang et al. 2020; Zhu and Li 2021; Liu and Zhang 2022), and the remaining species from the southern provinces of China. Despite advances in the taxonomic knowledge of the family, there are still many more genera and species to discover from southern China that have unusual morphological characteristics.

While working on the leptonetid fauna of the Jiangxi and Fujian provinces, southern China, we discovered and examined in detail eleven species including one known and ten new leptonetids. The goal of this paper is to formally describe the new species and to report the first species of *Falcileptoneta* Komatsu, 1970 from Jiangxi Province.

## ﻿Materials and methods

Specimens were examined using a Zeiss Stereo Discovery V12 stereomicroscope with a Zoom Microscope System. Both male palps and female genitalia were detached and examined in 80% ethanol, using a Zeiss Axio Scope A1 compound microscope with a KUY NICE CCD. The female genitalia were cleared in trypsin enzyme solution to dissolve soft tissues. For SEM photographs, specimens were dried under natural conditions, coated with gold using a small ion-sputtering apparatus ETD-2000, or without coating, and examined with a ZEISS EVO LS15 scanning electron microscope. Specimens including detached male palps and female genitalia were stored in 75% ethanol after examination. All the specimens are deposited in Animal Specimen Museum, Life Science of College, Jinggangshan University (**ASM-JGSU**).

To maintain uniformity of genitalia terminology within these genera, including *Falcileptoneta*, *Leptonetela*, *Longileptoneta*, and *Pararana*, the terms that are used are primarily from the Spider Anatomy Ontology on BioPortal (Ramírez and Michalik 2019). In the past, different terms have been used to refer to the same structure, and terms have been used incorrectly. Although some of these terms have synonyms in both males and females, the ones used here will hopefully become a standard for future studies of these genera, if applicable. Measurements were taken with the Axio Vision software (SE64 Rel. 4.8.3) and are given in millimeters. Leg measurements are given as total length (femur, patella, tibia, metatarsus, tarsus).

## ﻿Taxonomic account

### ﻿Family Leptonetidae Simon, 1890

**Genus *Falcileptoneta* Komatsu, 1970**

#### ﻿ Falcileptoneta monodactyla

Taxon classificationAnimaliaAraneaeLeptonetidae

(Yin, Wang & Wang, 1984)

D9F72651-5E01-5BF8-A1D0-56F84394662A

Figs 1 , 2

Leptoneta monodactyla Yin, Wang & Wang, 1984: 366, fig. 2a−d (holotype male, not examined; Hunan, Yanling); Song 1987: 104, fig. 67 (♂); Song et al. 1999: 51, fig. 21H−I (♂); Yin et al. 2012: 156, fig. 26a−d (♂); Liu et al. 2020: 3, figs 1A−E, 2A, B, 3A−C (♂).
Falcileptoneta monodactyla Wang, Li & Zhu, 2020: 689 (transferred from Leptoneta).

##### Material examined.

1 ♂, 26°30'41.64"N, 115°59'19.02"E, 346 m, Jinjing Cave, Cuiweifeng Forest Park, Ningdu County, Ganzhou City, Jiangxi Province, China, 23 January 2021, K. Liu, D. Zhao & Z. Meng leg. (Lep-3).

**Figure 1.**
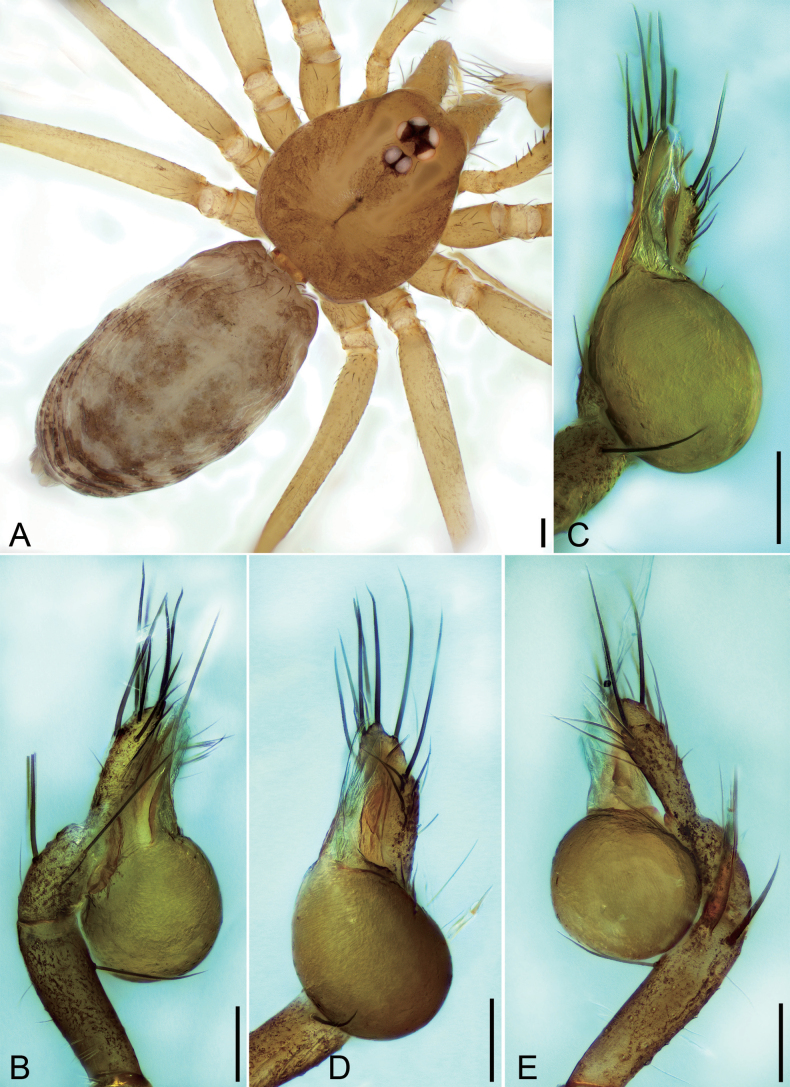
*Falcileptonetamonodactyla* (Yin, Wang & Wang, 1984), male **A** habitus, dorsal view **B** palp, prolateral view **C** same, ventral view **D** same, ventro-retrolateral view **E** same, retrolateral view. Scale bars: 0.1 mm.

**Figure 2.**
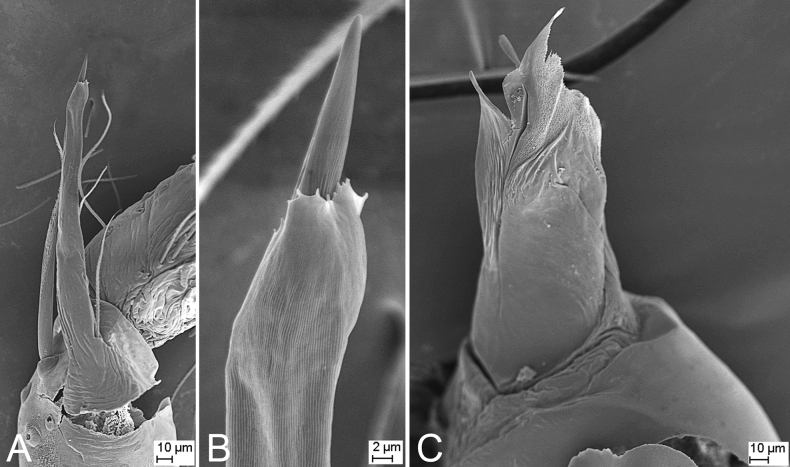
SEM micrographs of *Falcileptonetamonodactyla* (Yin, Wang & Wang, 1984), male right palp **A** tibial apophysis, retrolateral view **B** same, detail tip of tibial apophysis and spine, retrolateral view **C** tegular apophysis, ventral view.

##### Diagnosis and description.

See Liu et al. (2020) for both sexes.

##### Distribution.

Known from Jiangxi (new record) and Hunan (Liu et al. 2020), China (Fig. 29).

### ﻿Genus *Leptonetela* Kratochvíl, 1978

#### ﻿ Leptonetela dawu

Taxon classificationAnimaliaAraneaeLeptonetidae

Yao & Liu sp. nov.

2B7841B1-A5A4-5262-9F21-257763940615

https://zoobank.org/106D7200-E479-4F1C-A997-B1D976CC70E4

**Figure 3.**
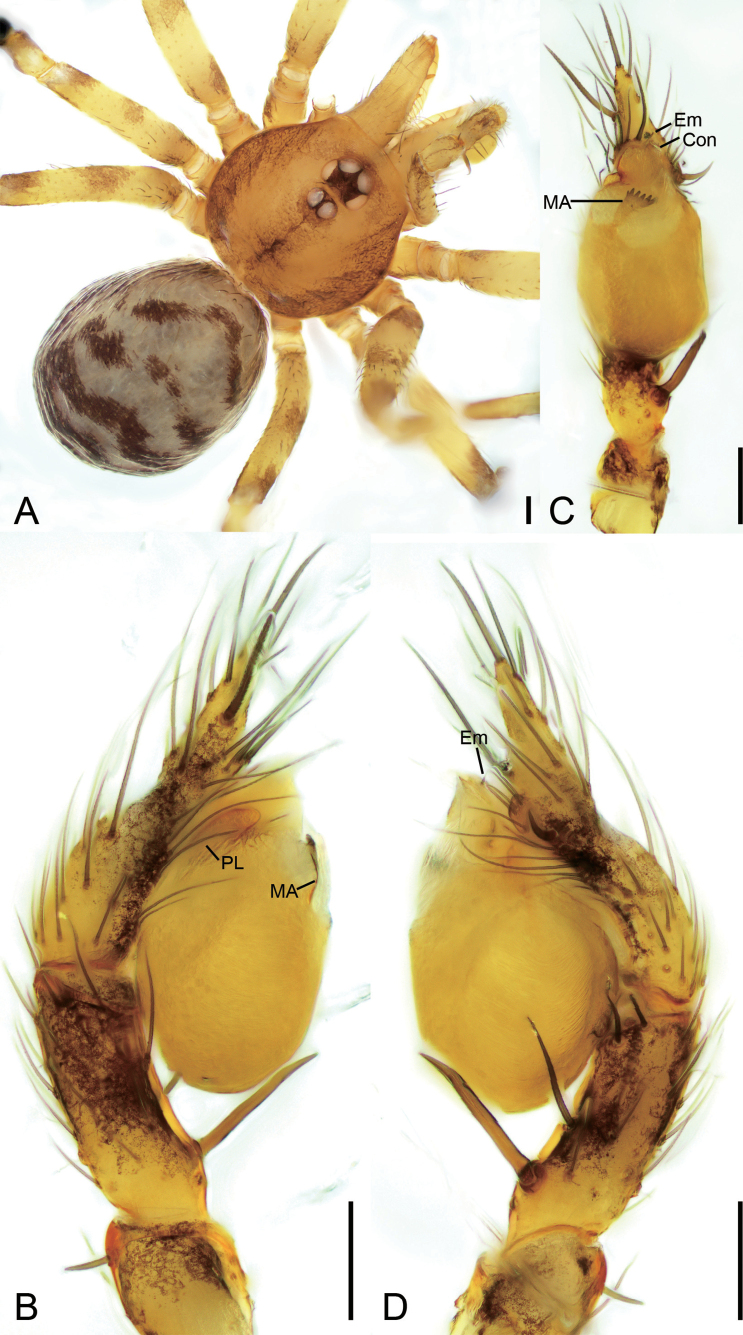
*Leptoneteladawu* sp. nov., male holotype **A** habitus, dorsal view **B** palp, prolateral view **C** same, ventral view **D** same, retrolateral view. Abbreviations: Con – conductor, Em – embolus, MA – medial apophysis, PL – prolateral lobe. Scale bars: 0.2 mm (**A**); 0.1 mm (**B–D**).

**Figure 4.**
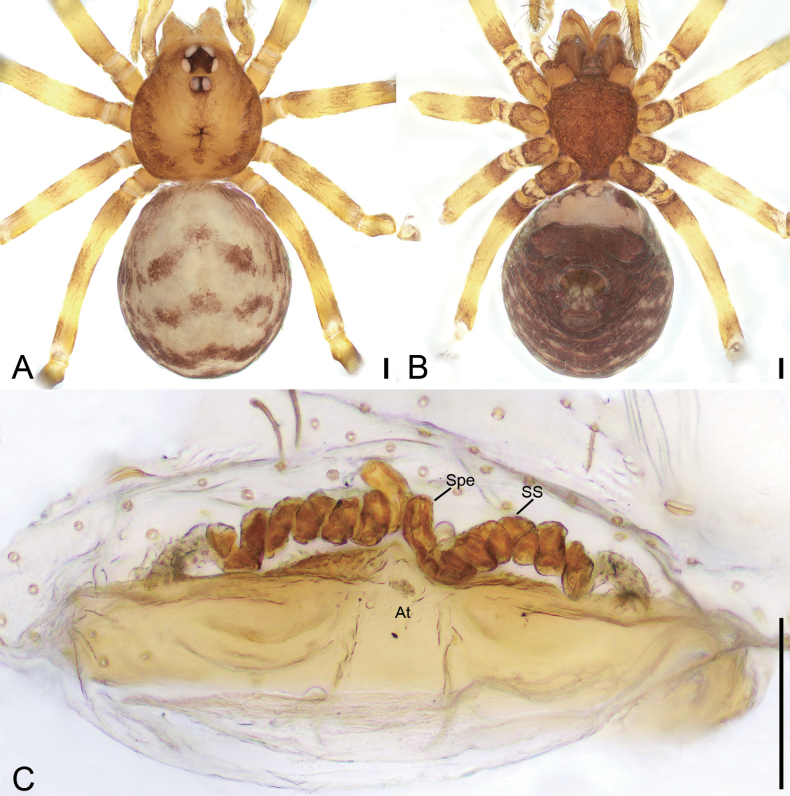
*Leptoneteladawu* sp. nov., female paratype **A** habitus, dorsal view **B** same, ventral view **C** vulva, dorsal view. Abbreviations: At – atrium, Spe – spermathecae, SS – spermathecae stalk. Scale bars: 0.1 mm.

Figs 3 , 4 , 8A–D Vernacular name: 大乌小弱蛛

##### Material examined.

***Holotype***: ♂, **China**: Jiangxi Province, Ji’an City, Qingyuan District, Donggu Town, Dawu Mountain, 26°40'48.69"N, 115°25'07.79"E, 1031 m, 25.X.2020, K. Liu, Y. Ying & S. Yuan leg. (Lep-8). ***Paratype***: 7 ♂, 2 ♀, the same data as the holotype (Lep-8).

##### Diagnosis.

The male of this species is similar to *Leptonetelasexdentata* Wang & Li, 2011 (see Wang and Li 2011: 15, figs 53A–D) in having a tongue-shaped prolateral lobe, but can be distinguished from it by dorsal habitus with obvious black-brown stripes (vs pale in *L.sexdentata*) and the foot-shaped median apophysis (vs square-shaped) (Figs 3, 8A–D). Females resemble that of *Leptonetelarudong* Wang & Li, 2017 (see Wang et al. 2017: 362, fig. 31C) in having a sub-rectangular atrium, but can be separated from it by the spermathecal stalk with seven spirals (vs six) and the slightly curved spermathecae (vs straight) (Fig. 4C).

##### Description.

**Male** (holotype). Habitus as in Fig. 3A. Total length 1.75. Carapace 0.82 long, 0.76 wide. Eye sizes and interdistances: ALE 0.09, PME 0.08, PLE 0.09; ALE–PME 0.12, PLE–PLE 0.11, PLE–PME 0.03; AER 0.17, PER 0.22. Clypeus 0.12 high. Chelicerae (Fig. 3A) with ten promarginal and five retromarginal teeth. Sternum shield-shaped, longer than wide, posterior end arch-shaped, smooth. Leg measurements: I 4.30 (1.13, 0.25, 1.21, 0.98, 0.73); II 3.32 (1.01, 0.19, 0.80, 0.74, 0.58); III 3.07 (1.00, 0.18, 0.68, 0.74, 0.47); IV 3.74 (0.97, 0.21, 1.06, 0.94, 0.56). Pedicel 0.12. Abdomen 0.98 long, 0.77 wide.

***Coloration*** (Fig. 3A). Carapace yellow to dark brown, with radial, dark brown, mottled markings on lateral margin and mottled stripes medially. Chelicerae, endites, labium, and sternum yellow-brown. Legs yellow, with distinct annulations. Abdomen pale to dark brown, with five dark chevron-shaped stripes.

***Palp*** (Figs 3B–D, 8A–D). Tibia with four long setae retrolaterally, the basal one thick, two short spines distally; cymbium with one thick, conspicuous spine prolaterally, one short, thick spine retrolaterally, and one long spine distally. Tip of bulb: prolateral lobe finger-like; median apophysis relatively long, foot-shaped, distal margin with ten teeth, the retrolateral one very large with blunt tip; conductor long, membranous, apically curved; embolus spine-like, very short, under the conductor.

**Female** (paratype). Total length 1.85. Carapace 0.89 long, 0.73 wide. Eye sizes and interdistances (Fig. 4A): ALE 0.09, PME 0.08, PLE 0.08; ALE–PME 0.10, PLE–PLE 0.11, PLE–PME 0.03; AER 0.16, PER 0.21. Clypeus 0.11 high. Chelicerae (Fig. 4B) with nine promarginal and five retromarginal teeth. Leg measurements: I (1.21, 0.28, other segments broken); II 3.19 (0.93, 0.21, 0.82, 0.79, 0.44); III (0.87, 0.24, other segments broken); IV (1.17, 0.18, other segments broken). Pedicel 0.05. Abdomen 1.22 long, 0.95 wide.

***Vulva*** (Fig. 4C). Internal genitalia with sub-rectangular atrium, finger-like spermathecae, and convoluted spermathecal stalk including six coils.

##### Distribution.

Known only from the type locality in Jiangxi Province, China (Fig. 29).

##### Etymology.

The name is taken from the type locality; noun in apposition.

#### ﻿ Leptonetela yuanhaoi

Taxon classificationAnimaliaAraneaeLeptonetidae

Yao & Liu sp. nov.

444A6E56-6F79-5D3E-8899-0407C315D49D

https://zoobank.org/74BE5C72-CB07-4A26-B274-BA45EA120254

**Figure 5.**
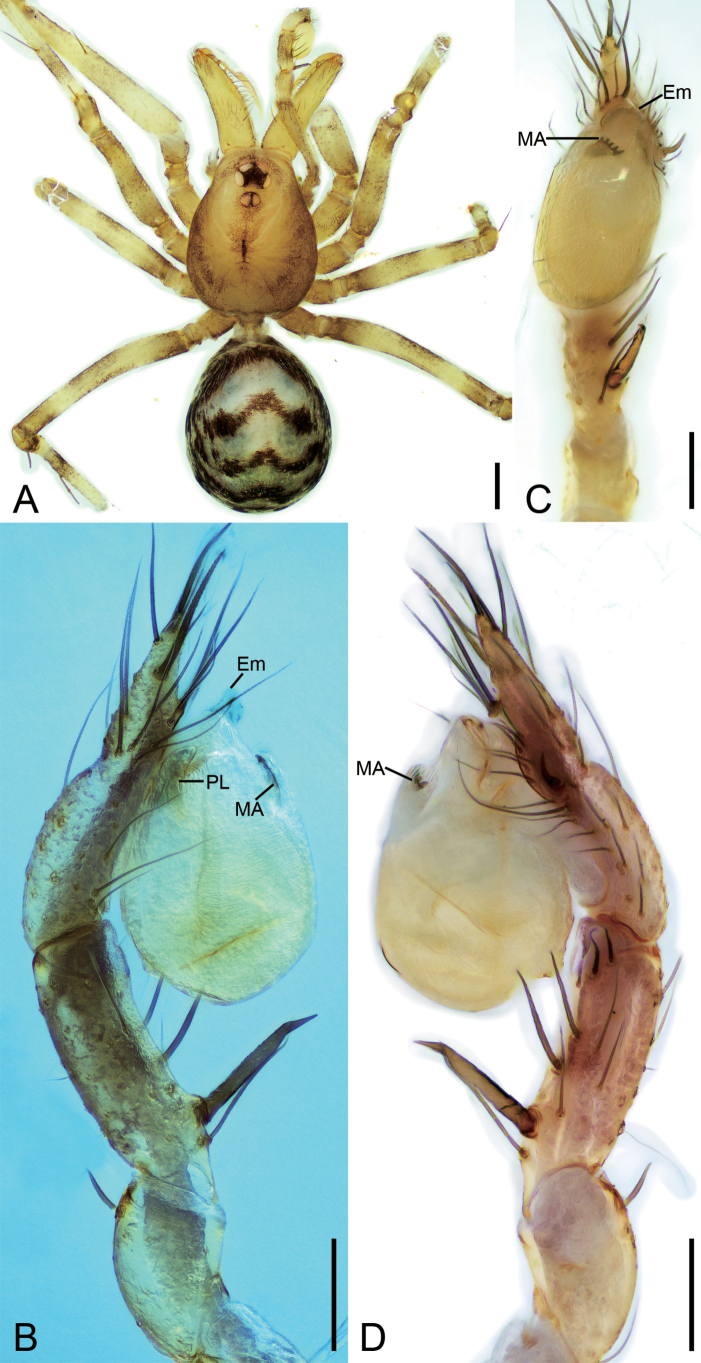
*Leptonetelayuanhaoi* sp. nov., male holotype **A** habitus, dorsal view **B** palp, prolateral view **C** same, ventral view **D** same, retrolateral view. Abbreviations: Em – embolus, MA – medial apophysis, PL – prolateral lobe. Scale bars: 0.1 mm.

**Figure 6.**
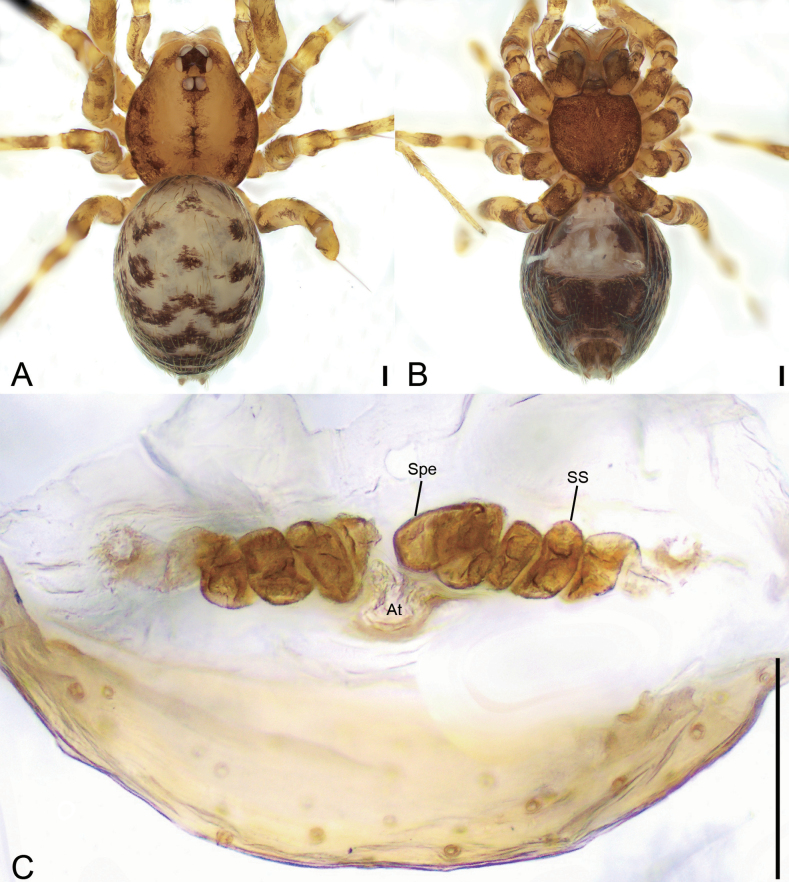
*Leptonetelayuanhaoi* sp. nov., female paratype **A** habitus, dorsal view **B** same, ventral view **C** vulva, dorsal view. Abbreviations: At – atrium, Spe – spermathecae, SS – spermathecae stalk. Scale bars: 0.1 mm.

Figs 5 , 6 , 8E–L Vernacular name: 渊浩小弱蛛

##### Material examined.

***Holotype***: ♂, **China**: Jiangxi Province, Ji’an City, Taihe County, Zhonglong Town, Zhonglong Village, Ziyao Mountain, 26°43'23.15"N, 115°13'31.70"E, 388 m, 28.X.2020, K. Liu, Y. Ying, K. Huang & S. Yuan leg. (Lep-7). ***Paratype***: 4 ♀, the same data as the holotype (Lep-7); 1 ♂, 26°42'58.10"N, 115°13'39.00"E, 206 m, other data as same as holotype (Lep-5); 1 ♂, 26°43'15.05"N, 115°13'37.85"E, 332 m other data same as holotype (Lep-6); 1 ♀, 26°43'05.30"N, 115°13'36.28"E, 228 m, other data same as holotype (Lep-1).

##### Diagnosis.

The male of this species is similar to that of *Leptonetelasexdentata* Wang & Li, 2011 (Wang and Li 2011: 15, fig. 53B–D) in having the tibia with a row of spines retrolaterally including one thick strong spine proximally and three thin spines, but can be separated from it by the tongue-shaped prolateral lobe (vs finger-like) and the median apophysis with narrow base (vs broad) and five teeth distally (under microscope) (vs six) (Figs 5B–D, 8E–L). The males also resemble that of *L.dawu* sp. nov. in having the conductor with curved apex and the spine-like embolus, but can be separated from it by the leaf-shaped median apophysis (vs foot-shaped) (Figs 5B–D, 8E–L). The female can be easily distinguished from *L.sexdentata* (Wang and Li 2011: 15, fig. 54C) by the transversely extended spermathecal stalk (vs directed anteromedially) with four regular spirals (vs irregular) (Fig. 6C).

##### Description.

**Male** (holotype). Habitus as in Fig. 5A. Total length 2.06. Carapace 0.91 long, 0.75 wide. Eye sizes and interdistances: ALE 0.09, PME 0.08, PLE 0.09; ALE–PME 0.13, PLE–PLE 0.10, PLE–PME 0.05; AER 0.18, PER 0.22. Clypeus 0.13 high. Chelicerae (Fig. 5A) with eight promarginal and six retromarginal teeth. Endites with several long spines anterolaterally. Sternum shield-shaped, nearly as long as wide, with dense scale-like surface, posterior end blunt. Leg measurements: I 4.60 (1.13, 0.32, 1.28, 1.04, 0.83); II 3.28 (0.75, 0.21, 0.96, 0.72, 0.64); III 2.94 (0.77, 0.21, 0.75, 0.81, 0.40); IV 3.82 (1.09, 0.15, 0.94, 0.98, 0.66); formula: I, IV, II, III. Pedicel 0.12. Abdomen 1.02 long, 0.89 wide.

***Coloration*** (Fig. 5A). Carapace yellow to dark brown, with dark radial stripes and mottled markings on lateral margin, and an oval dark brown band medially. Chelicerae yellow. Endites yellow to dark brown, mottled. Labium yellow to dark brown. Legs with dark annulations on each segment except tarsi. Abdomen with four dark chevron-shaped stripes.

***Palp*** (Figs 5B–D, 8E–L). Tibia with five long setae retrolaterally, the proximal one very thick, long, strong, spine-like; cymbium with one long conspicuous seta prolaterally, one short, strong, thick spine retrolaterally and one long spine distally. Tip of bulb: prolateral lobe tongue-like, relatively short; median apophysis leaf-shaped, distal margin with four to ten teeth, prolateral one very small, retrolateral one very large with triangular tip; conductor membranous, relatively broad, near the base of median apophysis, longer than median apophysis; embolus short, transparent, broad, slightly bending retrolaterally.

**Female** (paratype). Habitus as in Fig. 6A, B. Total length 1.70. Carapace 0.84 long, 0.71 wide. Eye sizes and interdistances: ALE 0.08, PME 0.08, PLE 0.08; ALE–PME 0.11, PLE–PLE 0.12, PLE–PME 0.04; AER 0.15, PER 0.20. Clypeus 0.10 high. Chelicerae (Fig. 6B) with nine promarginal and five retromarginal teeth. Endites with several long spines anterolaterally. Sternum (Fig. 6B) shield-shaped, nearly as long as wide, with dense scale-like surface, lateral margin thickened, posterior end blunt. Leg measurements: I 3.53 (1.00, 0.19, 1.05, 0.77, 0.52); II 2.80 (0.78, 0.20, 0.65, 0.59, 0.58); III 2.36 (0.71, 0.16, 0.56, 0.57, 0.36); IV 3.48 (0.97, 0.23, 0.89, 0.84, 0.55). Pedicel 0.06. Abdomen 1.06 long, 0.78 wide.

***Coloration*** (Fig. 6A, B). Darker than male.

***Vulva*** (Fig. 6C). Internal genitalia with sub-trapezoidal atrium, slightly swollen spermathecae. and convoluted spermathecal stalk including three coils.

##### Distribution.

Known only from the type locality in Jiangxi Province, China (Fig. 29).

##### Etymology.

The species is named after Mr Yuanhao Ying, who collected the type specimens.

##### Comments.

We compared the palps of the new species with that of the very similar species *L.dawu* sp. nov. several times. This similarity is probably because the males of these two species have very similar characters: tibial spines, cymbial spine, and embolus. The distance between Dawu Mountain and Ziyao Mountain is approximately 25 km (linear distances), which is very close. Despite the close distance, we consider them as different species based on the morphological differences listed. This hypothesis will be confirmed or rejected in the future when molecular data and analysis can be provided.

#### ﻿ Leptonetela zuojiashanensis

Taxon classificationAnimaliaAraneaeLeptonetidae

Yao & Liu sp. nov.

11797378-FBCF-5629-B7B6-088366426922

https://zoobank.org/1530D5D5-57E8-4792-83A2-7A3E4FC85771

**Figure 7.**
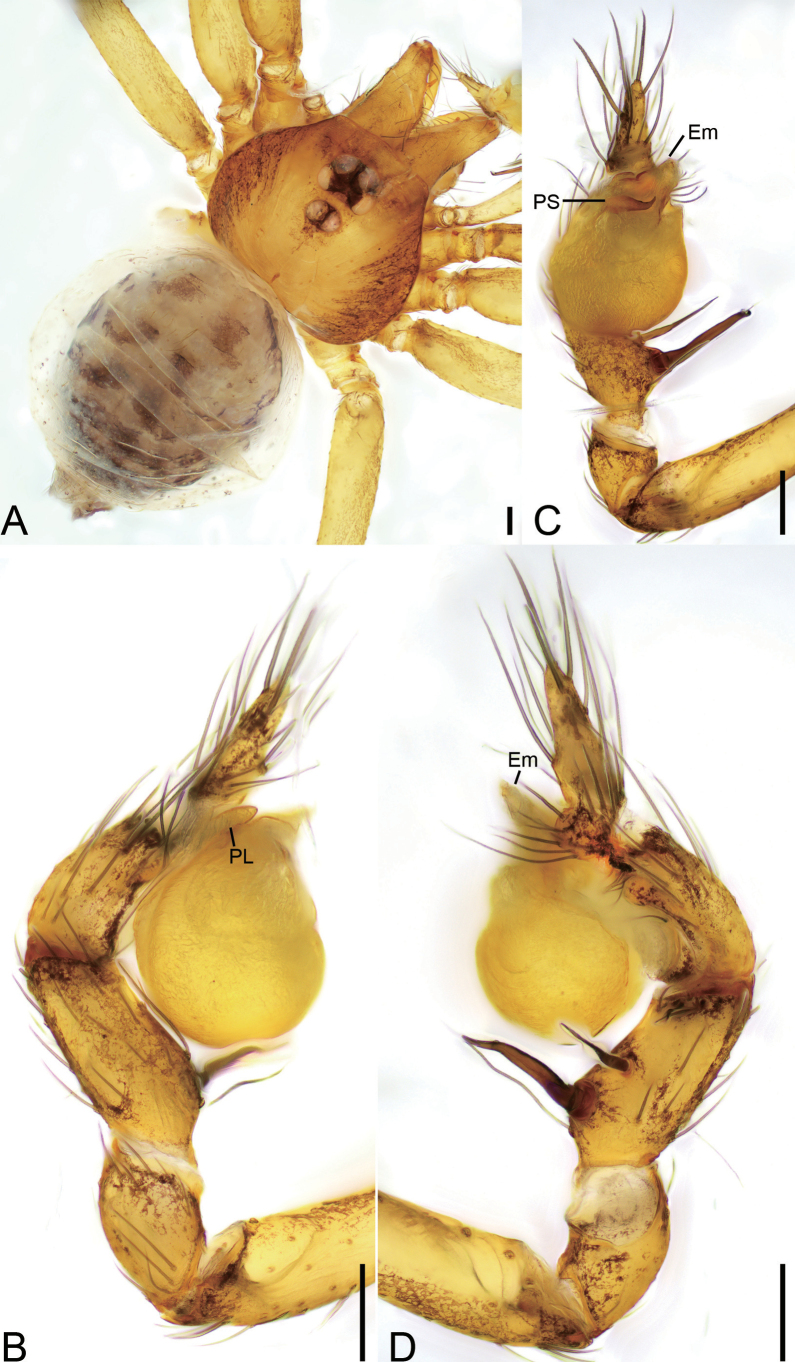
*Leptonetelazuojiashan* sp. nov., male holotype **A** habitus, dorsal view **B** palp, prolateral view **C** same, ventral view **D** same, retrolateral view. Abbreviations: Em – embolus, PL – prolateral lobe, PS – prolateral sclerite. Scale bars: 0.1 mm.

**Figure 8.**
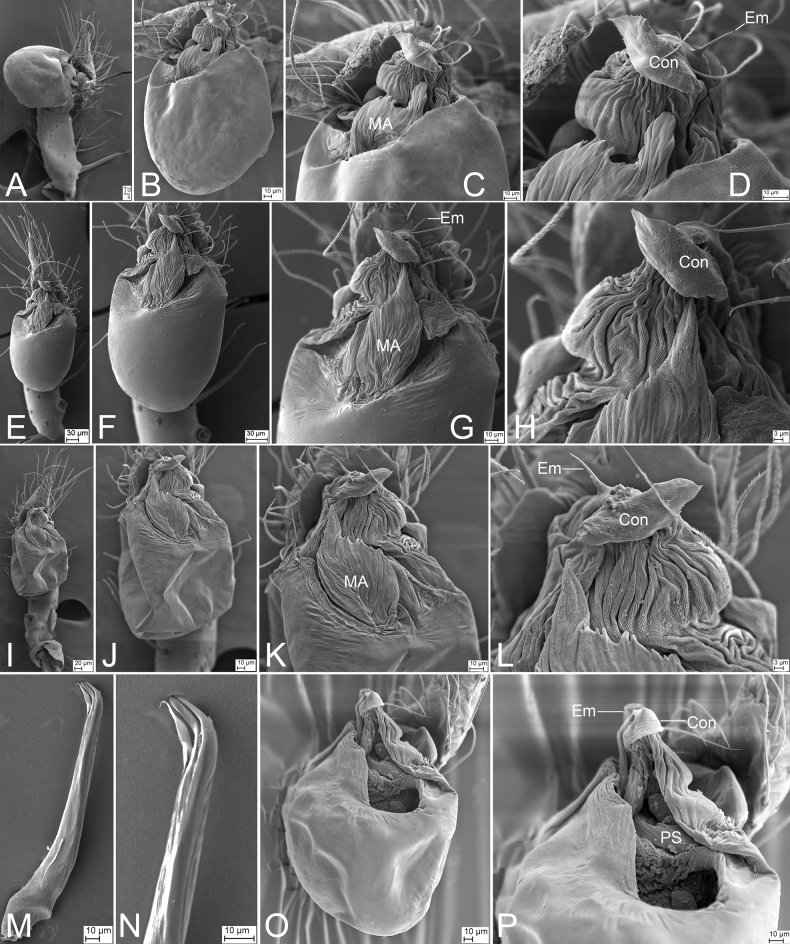
SEM micrographs of male palps, *Leptonetela* spp. **A***Leptoneteladawu* sp. nov., left palp, ventral view **B** same, ventral view **C** same, detail of tegular apophysis, ventral view **D** same, detail of conductor and embolus, ventral view **E***L.yuanhaoi* sp. nov., left palp, ventral view **F** same, ventral view **G** same, detail of tegular apophysis, ventral view **H** same, detail of conductor and embolus, ventral view **I***L.yuanhaoi* sp. nov., right palp, ventral view **J** same, ventral view **K** same, detail of tegular apophysis, ventral view **L** same, detail of conductor, ventral view **M***L.zuojiashanensis* sp. nov., right palp, tibial spine, prolateral view **N** same, detail of the tip, prolateral view **O** same, bulb, ventral view **P** same, detail of tegular apophysis, ventral view. Abbreviations: Con – conductor, Em – embolus, MA – medial apophysis, PS – prolateral sclerite.

Figs 7 , 8M–P Vernacular name: 左家山小弱蛛

##### Material examined.

***Holotype***: ♂, **China**: Jiangxi Province, Yichun City, Wanzai County, Luocheng Town, Jiulongshan Forest Park, Zuojiashan Village, 28°21'07.52"N, 114°30'27.58"E, 164 m, 6.II.2021, K. Liu, D. Zhao, Z. Meng, Z. He & W. Li leg. (Lep-4).

##### Diagnosis.

The male of this species is similar to that of *Leptonetelagubin* Wang & Li, 2017 (in Wang et al. 2017: 386, fig. 48B–D) in having the curved cymbium forming an angle of ca 100° with tibial axis and the horn-like prolateral sclerite, but can be separated from it by the tibia having two spines including one very thick and strong spine proximally (vs a row of spines and lacking a thick and strong spine) (Figs 7B–D, 8M–P). It also resembles that of *L.mengzongensis* Wang & Li, 2011 (Wang and Li 2011: 10, fig. 24B–D) in having the horn-like prolateral sclerite, but can be easily distinguished from it by the tibia with a thick and strong proximal spine (vs slender) (Figs 7B–D, 8M, N).

##### Description.

**Male** (holotype). Habitus as in Fig. 7A. Total length 2.08. Carapace 0.92 long, 0.89 wide. Eye sizes and interdistances: ALE 0.10, PME 0.09, PLE 0.11; ALE–PME 0.14, PLE–PLE 0.11, PLE–PME 0.05; AER 0.20, PER 0.24. Clypeus 0.13 high. Chelicerae (Fig. 7A) with seven promarginal and five retromarginal teeth. Sternum (Fig. 7A) hexagonal, longer than wide, posterior end blunt. Leg measurements: I 6.17 (1.71, 0.35, 1.66, 1.45, 1.00); II 4.93 (1.32, 0.32, 1.39, 1.11, 0.79); III 3.90 (0.96, 0.39, 1.01, 0.97, 0.57); IV (1.55, 0.29, other segments broken). Pedicel 0.10. Abdomen 1.06 long, 0.82 wide.

***Coloration*** (Fig. 7A). Carapace yellow to dark brown, with dark radial stripes and mottled markings on lateral margin. Chelicerae yellow. Endites yellow, with mottled dark spots. Labium, anterior part dark brown, posterior part yellow. Sternum dark brown, medially with a yellow stripe. Legs yellow to dark brown. Abdomen with three pairs of dark brown spots and three dark chevron-shaped stripes.

***Palp*** (Figs 7B–D, 8M–P). Tibia with two long spines retrolaterally, the basal one very thick and strong, with the trifurcate tip; cymbium lacking spine. Tip of bulb: prolateral lobe finger-like; prolateral sclerite relatively long, buffalo-horn-shaped; conductor membranous, narrowed, with curved tip; embolus short, indistinct, strongly bending dorsally.

**Female.** Unknown.

##### Distribution.

Known only from the type locality in Jiangxi Province, China (Fig. 29).

##### Etymology.

The name is taken from the type locality.

### ﻿Genus *Longileptoneta* Seo, 2015

#### ﻿ Longileptoneta guadunensis

Taxon classificationAnimaliaAraneaeLeptonetidae

Yao & Liu sp. nov.

96A46C53-3716-5498-9356-6D8C2229A5C6

https://zoobank.org/CFBD8259-59E8-41C9-8DF4-5FF173E53CCA

**Figure 9.**
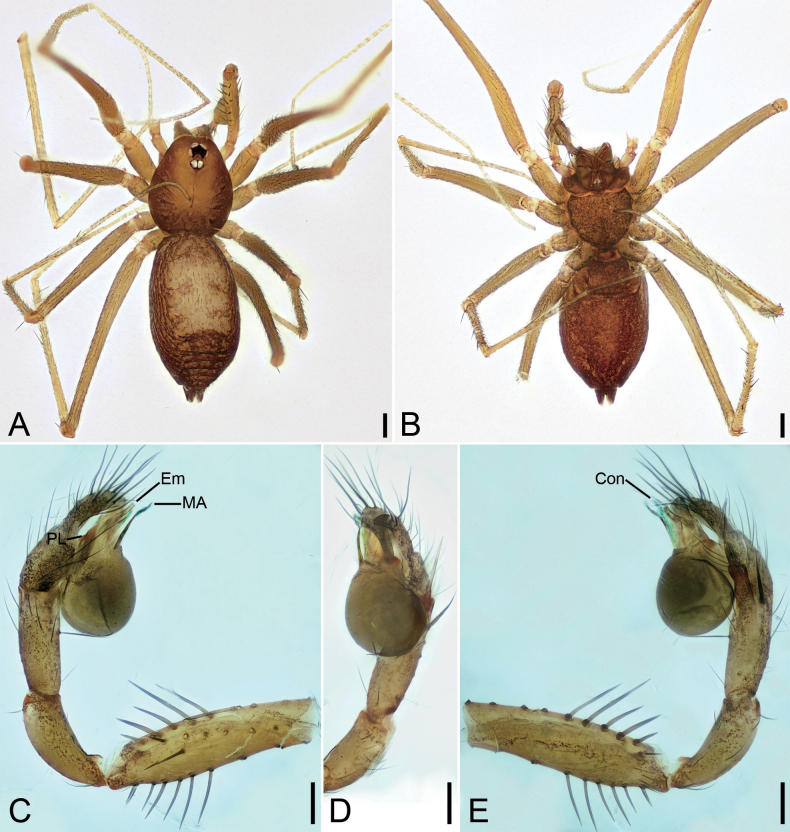
*Longileptonetaguadunensis* sp. nov., male holotype **A** habitus, dorsal view **B** same, ventral view **C** palp, prolateral view **D** same, ventral view, slightly retrolateral **E** same, retrolateral view. Abbreviations: Con – conductor, Em – embolus, MA – medial apophysis, PL – prolateral lobe. Scale bars: 0.2 mm (**A, B**); 0.1 mm (**C–E**).

**Figure 10.**
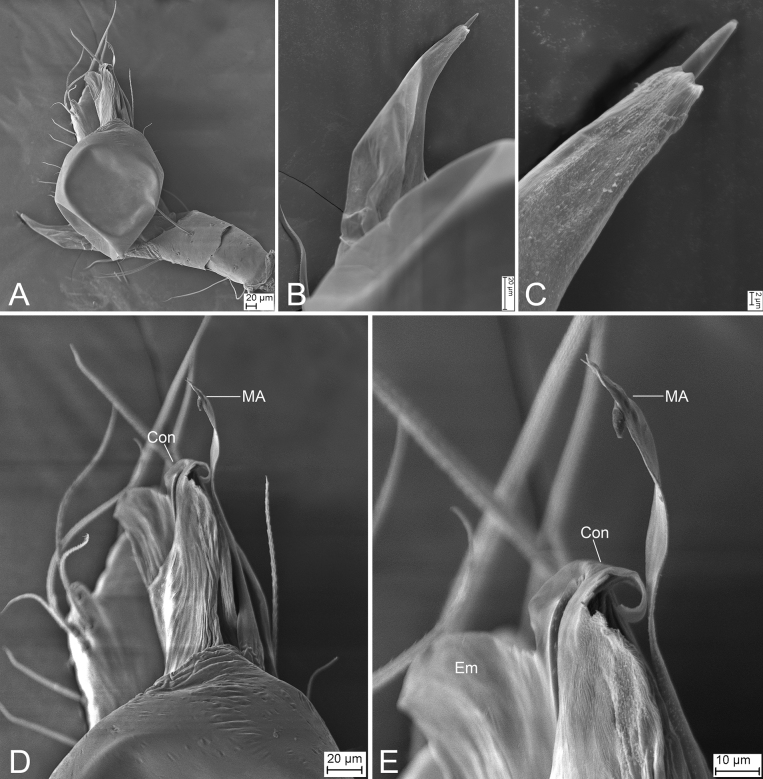
SEM micrographs of *Longileptonetaguadunensis* sp. nov., right palp, male holotype **A** ventral view **B** tibial apophysis, ventral view **C** detail of the tip of tibial apophysis, ventral view **D** detail of tegular apophysis, ventral view **E** same, ventral view. Abbreviations: Con – conductor, Em – embolus, MA – medial apophysis.

Figs 9 , 10 , 28A Vernacular name: 挂墩长弱蛛

##### Material examined.

***Holotype***: ♂, **China**: Fujian Province, Nanping City, Wuyishan County Level City, Xingcun Town, Guadun Village, 27°43'56.88"N, 117°39'30.29"E, 3.X.2023, Y. Yao, J. Gong & M. Wu leg. (Lep-13). ***Paratype***: 1 ♂, same data as the holotype (Lep-13).

##### Diagnosis.

This species is similar to that of *Longileptonetashenxian* Wang & Li, 2020 (in Wang et al. 2020: 698, fig. 12A–D) and *L.yamasakii* Ballarin & Eguchi, 2022 (Ballarin and Eguchi 2022: 373, figs 1C, 3A–C) in having the banded median apophysis with transparent tip, but can be distinguished from it by the carapace with six eyes (vs absent in *L.shenxian*), the tibia with one canine tooth-like apophysis armed with a short straight spine (vs one columnar apophysis, armed with one long, curved spine in *L.shenxian* and *L.yamasakii*) and the wedge-shaped prolateral lobe (vs mastoid in *L.shenxian* and sub-triangular in *L.yamasakii*) (Figs 9C–E, 10D, E).

##### Description.

**Male** (holotype). Habitus as in Fig. 9A, B. Total length 2.32. Carapace 0.86 long, 0.75 wide. Eye sizes and interdistances: ALE 0.08, PME 0.08, PLE 0.08; ALE–PME 0.11, PLE–PLE 0.12, PLE–PME 0.04; AER 0.14, PER 0.18. Clypeus 0.13 high. Chelicerae (Fig. 9B) with eight promarginal and six retromarginal teeth. Endites with several long spines laterally. Labium sub-rectangular, with several long setae. Sternum (Fig. 9B) shield-shaped, wider than long, posterior end blunt. Leg measurements: I 8.03 (2.08, 0.28, 2.63, 1.84, 1.20); II 4.73 (1.52, 0.23, 1.28, 0.97, 0.73); III 4.45 (1.17, 0.19, 1.29, 1.08, 0.72); IV 6.08 (1.51, 0.25, 1.98, 1.57, 0.77). Pedicel 0.05. Abdomen 1.42 long, 0.83 wide.

***Coloration*** (Fig. 9A, B). Carapace yellow to black-brown, with radial dark stripes near submargin. Chelicerae, endites and labium brown. Sternum yellow to dark brown, mottled. Legs yellow, with sparse mottling. Abdomen dorsally yellowish, mottled in lateral and posterior parts; venter brown, mottled.

***Palp*** (Figs 9C–E, 10). Femur with three rows of long strong spines ventrally, dorsally, and prolaterally; patella without spine; tibia with two spines: one canine tooth-like apophysis, armed with a short straight spine subapically, and a thick and long spine near the base of anterior one. Cymbium with a constriction subapically. Tip of bulb: one long banded median apophysis, distally furcate; prolateral lobe wedge-shaped, large; conductor membranous, anteriorly curved. Embolus indistinct, with broad base.

**Female.** Unknown.

##### Distribution.

Known only from the type locality in Fujian Province, China (Fig. 29).

##### Etymology.

The name is taken from the type locality.

#### ﻿ Longileptoneta huboliao

Taxon classificationAnimaliaAraneaeLeptonetidae

Yao & Liu sp. nov.

80F3416C-8F2D-55A2-92CA-56D39373707D

https://zoobank.org/28EAA35E-4067-43E3-B40F-10B70FF5A93B

**Figure 11.**
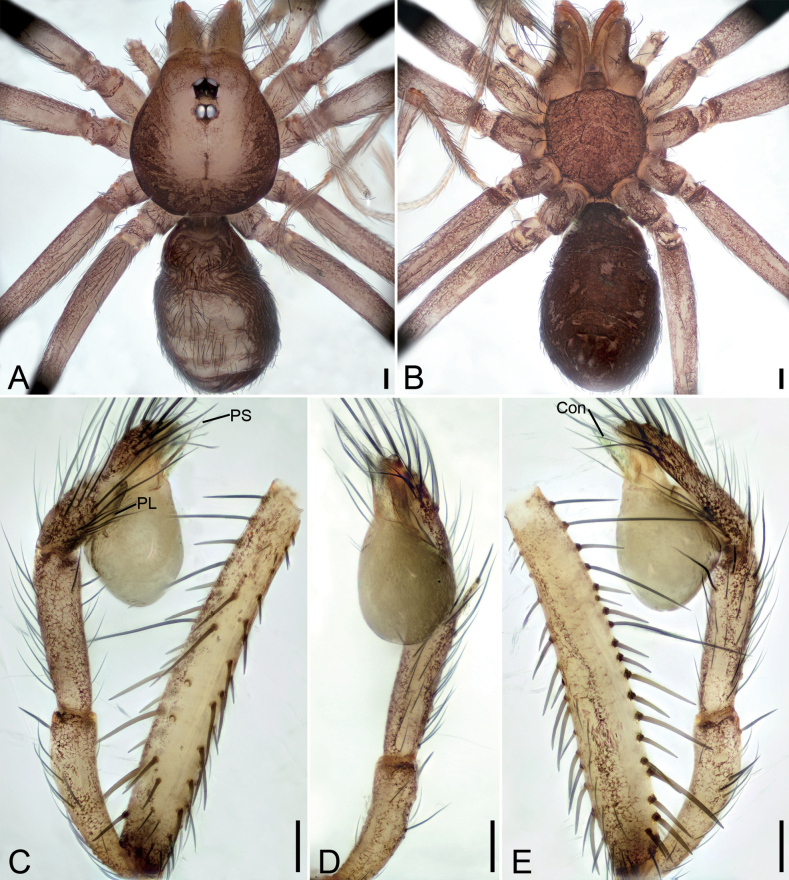
*Longileptonetahuboliao* sp. nov., male holotype **A** habitus, dorsal view **B** same, ventral view **C** palp, prolateral view **D** same, ventro-retrolateral view **E** same, retrolateral view. Abbreviations: Con – conductor, PL – prolateral lobe, PS – prolateral sclerite. Scale bars: 0.1 mm.

**Figure 12.**
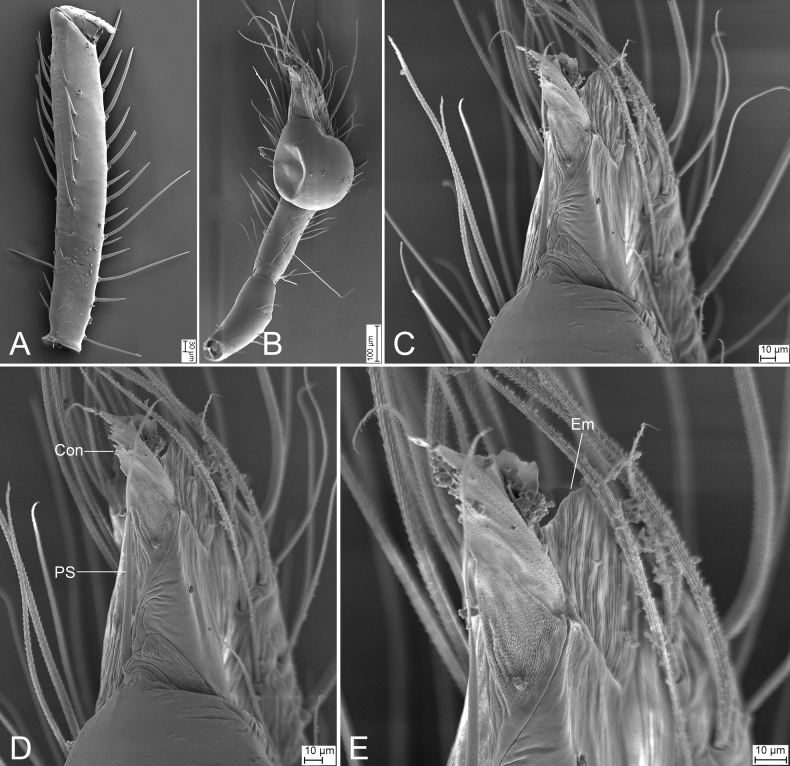
SEM micrographs of *Longileptonetahuboliao* sp. nov., male palp **A** femur, prolateral view **B** palp, ventral view **C** detail of tegular apophysis, ventral view **D** same, ventral view **E** detail of embolus, ventral view. Abbreviations: Con – conductor, Em – embolus, PS – prolateral sclerite.

**Figure 13.**
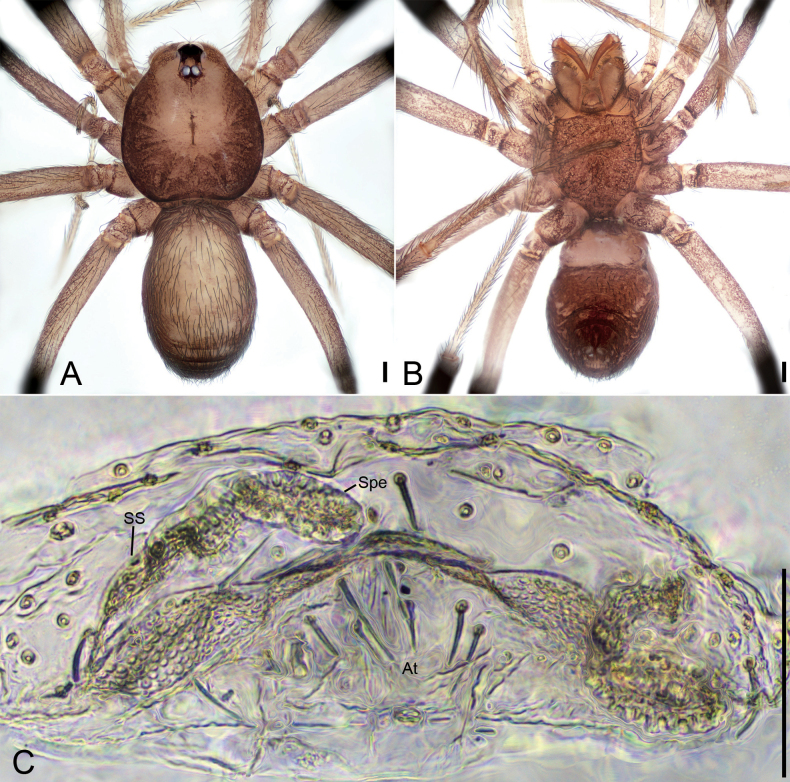
*Longileptonetahuboliao* sp. nov., female paratype **A** habitus, dorsal view **B** same, ventral view **C** vulva, dorsal view. Abbreviations: At – atrium, Spe – spermathecae, SS – spermathecae stalk. Scale bars: 0.1 mm.

Figs 11 , 12 , 13 , 28B Vernacular name: 虎伯寮长弱蛛

##### Material examined.

***Holotype***: ♂, **China**: Fujian Province, Zhangzhou City, Nanjing County, Huboliao Nature Reserve, Huboliao area, 24°31'2.88"N, 117°14'53.47"E, 08.XI.2023, Y. Yao, J. Gong, R. Zhao & M. Wu leg. (Lep-15). ***Paratype***: 1 ♀, the same data as the holotype (Lep-15); 1 ♂, 24°31'20.98"N, 117°17'32.01"E, 09.XI.2023, Y. Yao, J. Gong, R. Zhao & M. Wu leg. (Lep-15).

##### Diagnosis.

The male of this species is similar to that of *Longileptonetashenxian* Wang & Li, 2020 (in Wang et al. 2020: 698, fig. 12A–D) in having the bulb with an extruded coniform anterior part and three rows of spines, but can be distinguished from it by the carapace with six eyes (vs absent), the tibia with one long columnar apophysis armed with a long spine (vs one short columnar apophysis, armed with one long, curved spine) and the long needle-like prolateral sclerite (vs thick) (Figs 11, 12). The female can be easily separated from *L.shenxian* (Wang et al. 2020: 698, fig. 13C) by the bell-shaped atrium, but can be separated by the carapace with six eyes (vs absent) and the distal spermathecal stalk lacking a coil (vs present) (Fig. 13).

##### Description.

**Male** (holotype). Habitus as in Fig. 11A, B. Total length 2.11. Carapace 0.96 long, 0.82 wide. Eye sizes and interdistances: ALE 0.08, PME 0.07, PLE 0.07; ALE–PME 0.12, PLE–PLE 0.11, PLE–PME 0.05; AER 0.15, PER 0.19. Clypeus 0.18 high. Chelicerae (Fig. 11B) with nine promarginal and eight retromarginal teeth. Endites with several long setae laterally. Labium sub-rectangular, anteriorly with more than two pairs of strong setae. Sternum (Fig. 11B) shield-shaped, slightly longer than wide, with several long setae on the surface, posterior end blunt. Leg measurements: I 8.04 (1.93, 0.31, 2.55, 2.18, 1.07); II 5.58 (1.54, 0.28, 1.62, 1.34, 0.80); III 4.20 (1.00, 0.27, 1.29, 1.09, 0.55); IV 6.23 (1.76, 0.29, 2.05, 1.57, 0.56). Pedicel 0.11. Abdomen 1.03 long, 0.67 wide.

***Coloration*** (Fig. 11A, B). Carapace reddish to black-brown, with radial dark brown stripes along submargin. Chelicerae and endites reddish to dark brown. Labium brown. Sternum brown, mottled. Legs reddish to black-brown, mottled. Abdomen medial part reddish, other parts dark brown, mottled; venter dark brown, mottled.

***Palp*** (Figs 11C–E, 12). Femur with three rows of short strong spines ventrally, dorsally, and prolaterally; patella lacking spine; tibia with one long columnar apophysis, armed with a long straight spine. Tip of bulb: prolateral lobe willow leaf-shaped; prolateral sclerite long, needle-like; conductor membranous, with serrate tip. Embolus with blunt tip, shorter than prolateral sclerite.

**Female** (paratype). Habitus as in Fig. 13A, B. Total length 1.77. Carapace 0.89 long, 0.76 wide. Eye sizes and interdistances: ALE 0.08, PME 0.07, PLE 0.07; ALE–PME 0.12, PLE–PLE 0.1, PLE–PME 0.05; AER 0.15, PER 0.18. Clypeus 0.14 high. Chelicerae (Fig. 13B) with nine promarginal and eight retromarginal teeth. Endites with several long spines anterolaterally. Sternum (Fig. 13B) shield-shaped, nearly as long as wide, with dense scale-like surface, lateral margin thickened, posterior end blunt. Leg measurements: I 6.25 (1.90, 0.23, 1.97, 1.53, 0.62); II 5.19 (1.44, 0.26, 1.52, 1.22, 0.75); III 4.18 (1.13, 0.28, 1.24, 0.89, 0.64); IV 6.38 (1.66, 0.28, 2.07, 1.58, 0.79). Pedicel 0.04. Abdomen 0.84 long, 0.59 wide.

***Vulva*** (Fig. 13C). Internal genitalia with semicircle atrium, oval spermathecae, and convoluted spermathecal stalk including three coils.

##### Note.

The right spermathecal stalk and spermathecae were extruded deformation after covering cover slip when we took a photo under microscope.

##### Distribution.

Known only from the type locality in Fujian Province, China (Fig. 29).

##### Etymology.

The name is taken from the type locality, noun in apposition.

#### ﻿ Longileptoneta jiaxiani

Taxon classificationAnimaliaAraneaeLeptonetidae

Yao & Liu sp. nov.

840F31EA-5433-5B1E-84E1-6A7BF2AA196D

https://zoobank.org/1D1C5F43-5FFE-4C15-9987-E304121393C9

**Figure 14.**
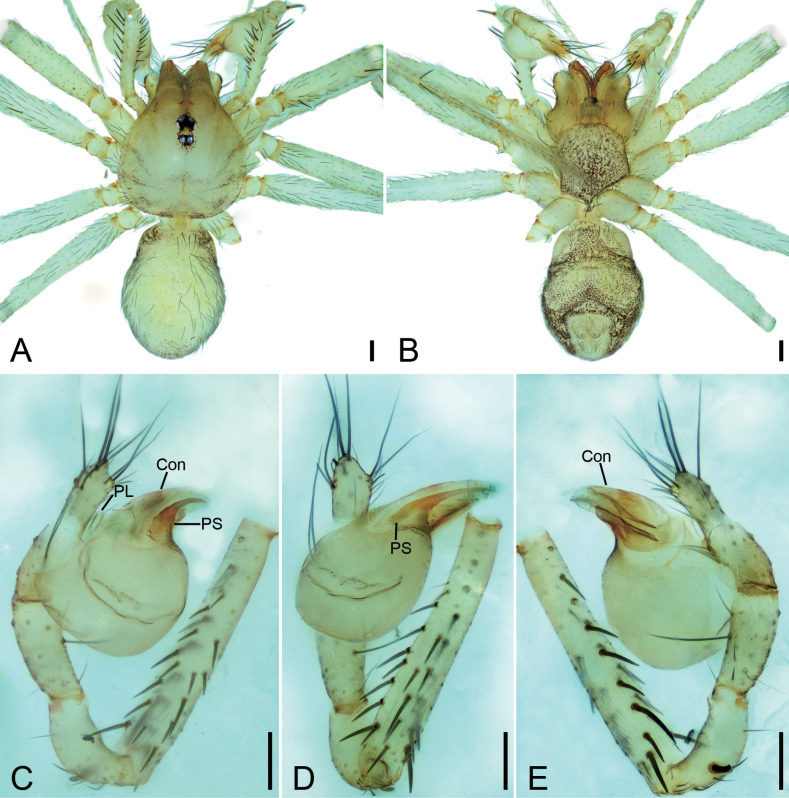
*Longileptonetajiaxiani* sp. nov., male palp, holotype **A** habitus, dorsal view **B** same, ventral view **C** palp, prolateral view **D** same, ventral view, slightly prolateral **E** same, retrolateral view. Abbreviations: Con – conductor, PL – prolateral lobe, PS – prolateral sclerite. Scale bars: 0.1 mm.

**Figure 15.**
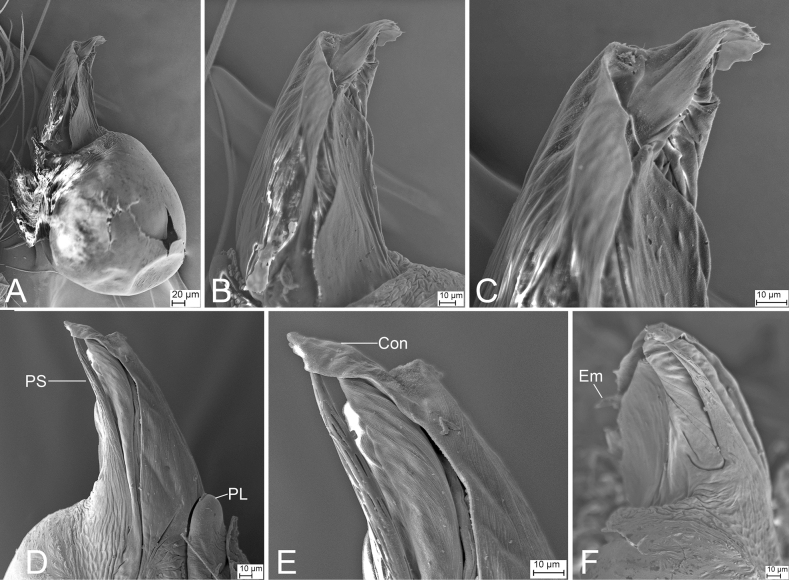
SEM micrographs of *Longileptonetajiaxiani* sp. nov., right male palp, holotype **A** right palp, retrolateral view **B** same, detail of tegular apophysis, retrolateral view **C** same, retrolateral view **D** same, detail of tegular apophysis, prolateral view, **E** same, detail of conductor, prolateral view **F** same, detail of embolus, ventral view. Abbreviations: Con – conductor, Em – embolus, PL – prolateral lobe, PS – prolateral sclerite.

**Figure 16.**
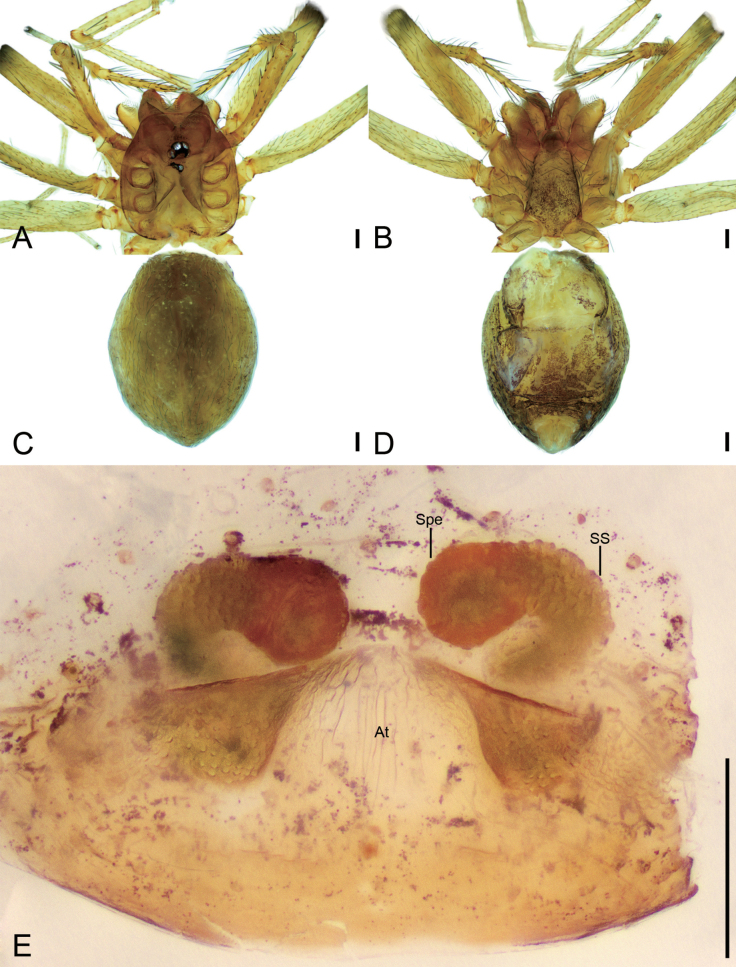
*Longileptonetajiaxiani* sp. nov., female paratype **A** carapace, dorsal view **B** same ventral view **C** abdomen, dorsal view **D** same, ventral view **E** vulva, dorsal view. Abbreviations: At – atrium, Spe – spermathecae, SS – spermathecae stalk. Scale bars: 0.1 mm.

Figs 14 , 15 , 16 , 28C Vernacular name: 嘉贤长弱蛛

##### Material examined.

***Holotype***: ♂, **China**: Fujian Province, Fuzhou City, Cangshan District, Jinshan campus in Fujian Agriculture and Forestry University, 26°2'21.12"N, 119°19'56.66"E, 29.IV.2023, Y. Yao, J. Gong & M. Wu leg. (Lep-10). ***Paratype***: 1 ♀, the same data as the holotype (Lep-10).

##### Diagnosis.

The male of this species is similar to that of *L.shenxian* Wang & Li, 2020 (in Wang et al. 2020: 698, fig. 12A–D) in having the bulb with an extruded coniform anterior part and the spine-like prolateral sclerite, but can be distinguished from it by the carapace with six eyes (vs absent) and the patella with a very strong and thick spine (vs absent), and the hook-shaped embolus (vs the narrowed lamellar embolus) (Figs 14C–E, 15). The female resembles *L.shenxian* (Wang et al. 2020: 698, fig. 13A–C) in having a bell-shaped atrium, but can be separated by the carapace with eyes (vs lacking) and the C-shaped spermathecal stalk (vs S-shaped) (Fig. 16).

##### Description.

**Male** (holotype). Habitus as in Fig. 14A, B. Total length 1.46. Carapace 0.68 long, 0.65 wide. Eye sizes and interdistances: ALE 0.05, PME 0.04, PLE 0.05; ALE–PME 0.07, PLE–PLE 0.07, PLE–PME 0.03; AER 0.10, PER 0.12. Clypeus 0.13 high. Chelicerae (Fig. 14B) with eight promarginal and five retromarginal teeth. Endites with several long spines laterally and seven leaf-shaped setae. Labium sub-rectangular, anterolaterally with two pairs of strong setae and anteriorly with eight setae. Sternum (Fig. 14B) shield-shaped, longer than wide, with dense setae laterally, posterior end blunt. Leg measurements: I 5.23 (1.34, 0.19, 1.61, 1.29, 0.80); II (1.42, 0.28, other segments broken); III 4.52 (1.19, 0.29, 1.15, 1.08, 0.81); IV (1.68, 0.24, other segments broken). Pedicel 0.06. Abdomen 0.73 long, 0.53 wide.

***Coloration*** (Fig. 14A, B). Carapace yellowish to black, with radial dark stripes submedially and mottled markings on lateral margin. Chelicerae yellow to dark brown. Endites yellow. Labium yellow. Sternum yellow to black, mottled. Legs yellow. Abdomen, dorsally yellow, mottled in anterior and posterior parts; venter mottled.

***Palp*** (Figs 14C–E, 15). Femur with four rows of short strong spines ventrally, dorsally, and prolaterally; patella with one thick, strong spine proximally; tibia lacking spine and apophysis. Cymbium with a distinct constriction medially. Tip of bulb: one spine-like prolateral sclerite; prolateral lobe lamellar; conductor membranous, with banded tip, slightly shorter than prolateral sclerite. Embolus indistinct, wrapping with conductor, hook-shaped.

**Female** (paratype). Habitus as in Fig. 16A–D. Total length 1.92. Carapace 0.78 long, 0.67 wide. Eye sizes and interdistances: ALE 0.05, PME 0.04, PLE 0.05; ALE–PME 0.09, PLE–PLE 0.09, PLE–PME 0.05; AER 0.10, PER 0.15. Clypeus 0.15 high. Chelicerae (Fig. 16B) with eight promarginal and seven retromarginal teeth. Endites with several long spines anterolaterally. Sternum (Fig. 16B) shield-shaped, nearly as long as wide, with dense scale-like surface, lateral margin thickened, posterior end blunt. Leg measurements: I (1.40, 0.29, 1.69, other segments broken); II 4.33 (1.21, 0.29, 1.23, 0.99, 0.61); III 3.41 (0.98, 0.13, 0.93, 0.81, 0.56); IV (1.34, 0.21, 1.48, 1.16, other segments broken). Abdomen 1.14 long, 0.80 wide.

***Vulva*** (Fig. 16E). Internal genitalia with sub-trapezoidal atrium, slightly swollen spermathecae, and convoluted spermathecal stalk including three coils.

##### Distribution.

Known only from the type locality in Fujian Province, China (Fig. 29).

##### Etymology.

The species is named after Mr Jiaxian Gong, who collected the type specimens.

#### ﻿ Longileptoneta letuensis

Taxon classificationAnimaliaAraneaeLeptonetidae

Yao & Liu sp. nov.

E943468F-DF83-5F2D-A306-7106FB9AC8EF

https://zoobank.org/AAB005C2-29DD-462B-94F6-DB83706C1911

**Figure 17.**
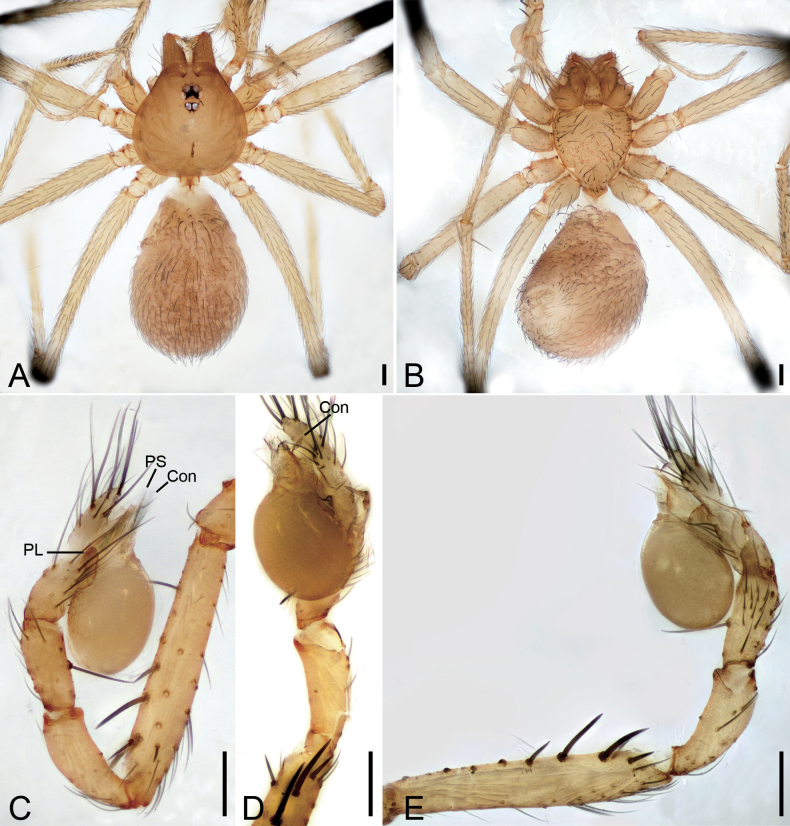
*Longileptonetaletuensis* sp. nov., male holotype **A** habitus, dorsal view **B** same, ventral view **C** palp, prolateral view **D** same, ventral view, slightly retrolateral **E** same, retrolateral view. Abbreviations: Con – conductor, PL – prolateral lobe, PS – prolateral sclerite. Scale bars: 0.1 mm.

**Figure 18.**
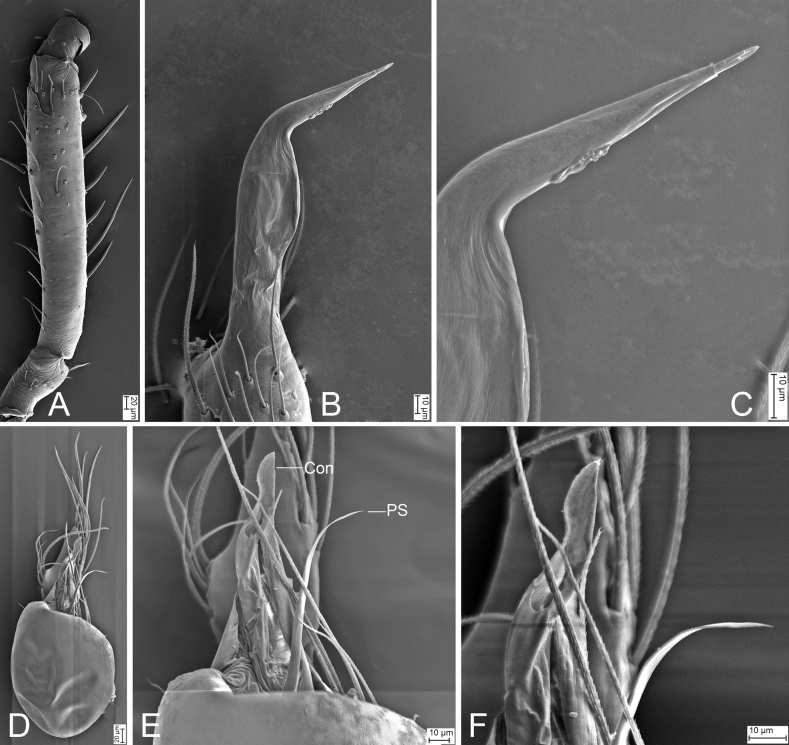
SEM micrographs of *Longileptonetaletuensis* sp. nov., male right palp **A** femur, retrolateral view **B** tibial apophysis, retrolateral view **C** detail of the tip of tibial apophysis, retrolateral view **D** bulb, ventral view **E** same, detail of tegular apophysis, ventral view **F** same, detail of conductor, ventral view. Abbreviations: Con – conductor, PS – prolateral sclerite.

Figs 17 , 18 , 28D Vernacular name: 乐土长弱蛛

##### Material examined.

***Holotype***: ♂, **China**: Fujian Province, Zhangzhou City, Nanjing County, Huboliao Nature Reserve, Letu Rainforest area, 24°54'11.82"N, 117°13'15.3"E, 11.XI.2023, Y. Yao, J. Gong, R. Zhao & M. Wu leg. (Lep-16).

##### Diagnosis.

The male of this species can be easily distinguished from other members of this genus by the very large curved tibial apophysis armed with a short spine-like tip (Fig. 17E).

##### Description.

**Male** (holotype). Habitus as in Fig. 17A, B. Total length 1.78. Carapace 1.04 long, 0.64 wide. Eye sizes and interdistances: ALE 0.04, PME 0.04, PLE 0.04; ALE–PME 0.07, PLE–PLE 0.07, PLE–PME 0.03; AER 0.09, PER 0.12. Clypeus 0.06 high. Chelicerae (Fig. 17B) with eight promarginal and six retromarginal teeth. Endites with several long spines laterally. Sternum (Fig. 17B) shield-shaped, nearly as long as wide, with abundant long setae on surface, posterior end blunt. Leg measurements: I 4.75 (1.34, 0.24, 1.46, 1.14, 0.57); II 3.60 (0.95, 0.24, 1.10, 0.86, 0.45); III 2.80 (0.85, 0.22, 0.79, 0.58, 0.36); IV 4.3 (1.19, 0.24, 1.28, 1.02, 0.57). Pedicel 0.08. Abdomen 0.67 long, 0.66 wide.

***Coloration*** (Fig. 17A, B). Carapace yellowish, with radial yellow stripes submarginally. Chelicerae, endites and labium yellow. Sternum yellowish, mottled. Legs yellowish. Abdomen dorsally yellowish, with three pairs of indistinct reddish spots; venter mottled.

***Palp*** (Figs 17C–E, 18). Femur with two rows of short strong spines ventrally and prolaterally; patella without spine; tibia with a very long curved apophysis, distally armed with a short straight spine, longer than tibia. Cymbium with a distinct constriction medially. Tip of bulb: one long flagelliform prolateral sclerite; prolateral lobe oval; conductor membranous, medially with a groove. Embolus indistinct.

**Female.** Unknown.

##### Distribution.

Known only from the type locality in Fujian Province, China (Fig. 29).

##### Etymology.

The name is taken from the type locality.

#### ﻿ Longileptoneta renzhouensis

Taxon classificationAnimaliaAraneaeLeptonetidae

Yao & Liu sp. nov.

67C4DD3D-A9D8-5DAD-8BED-3267D8E3E1FF

https://zoobank.org/EA2C7512-B01E-4021-934F-8E40EE47555F

**Figure 19.**
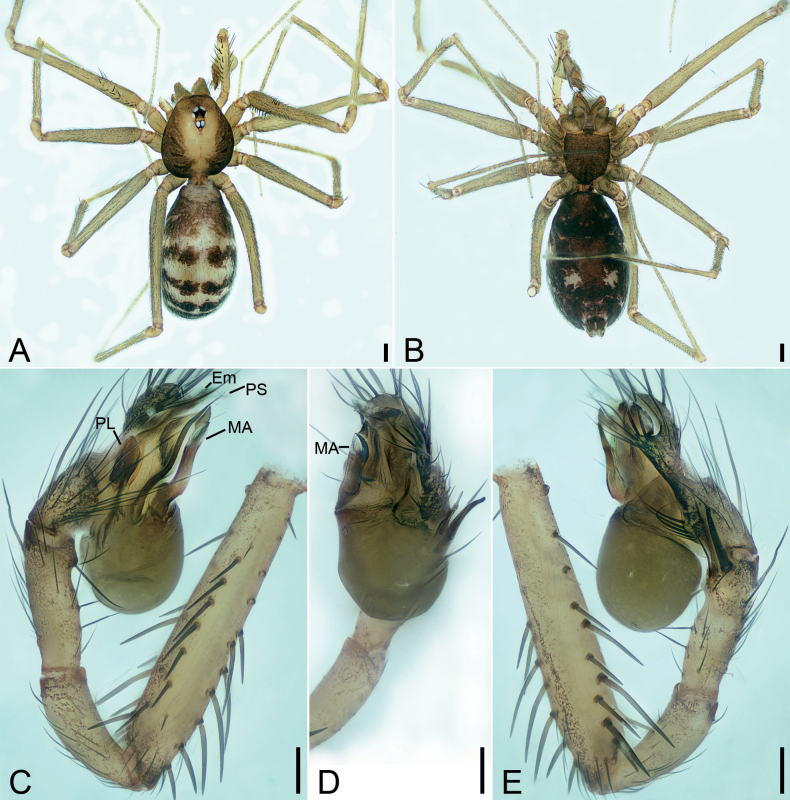
*Longileptonetarenzhouensis* sp. nov., male holotype **A** habitus, dorsal view **B** B habitus, ventral view **C** palp, prolateral view **D** same, ventral view **E** same, retrolateral view. Abbreviations: Em – embolus, MA – medial apophysis, PL – prolateral lobe, PS – prolateral sclerite. Scale bars: 0.2 mm (**A, B**); 0.1 mm (**C–E**).

**Figure 20.**
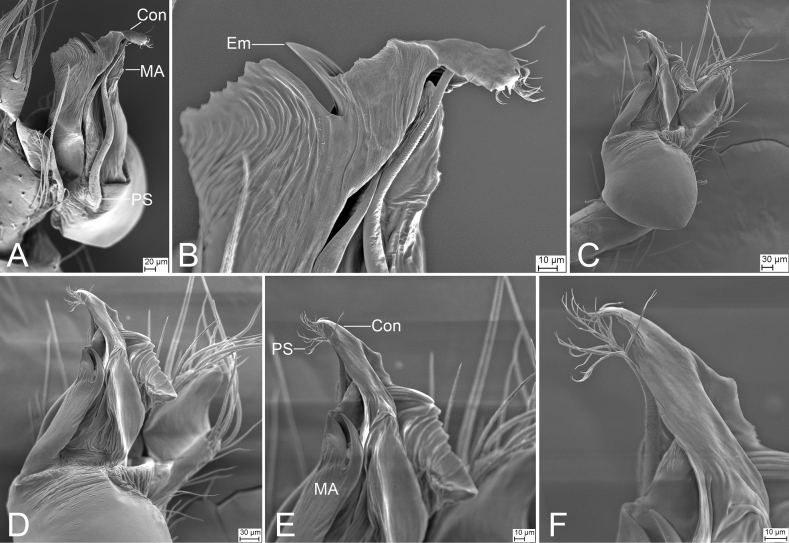
SEM micrographs of *Longileptonetarenzhouensis* sp. nov., male palp **A** palp, prolateral view **B** detail of tegular apophysis, prolateral view **C** palp, ventral view **D** same, detail of tegular apophysis, ventral view **E** same, ventral view **F** same, detail of conductor and prolateral sclerite, ventral view. Abbreviations: Con – conductor, Em – embolus, MA – medial apophysis, PS – prolateral sclerite.

**Figure 21.**
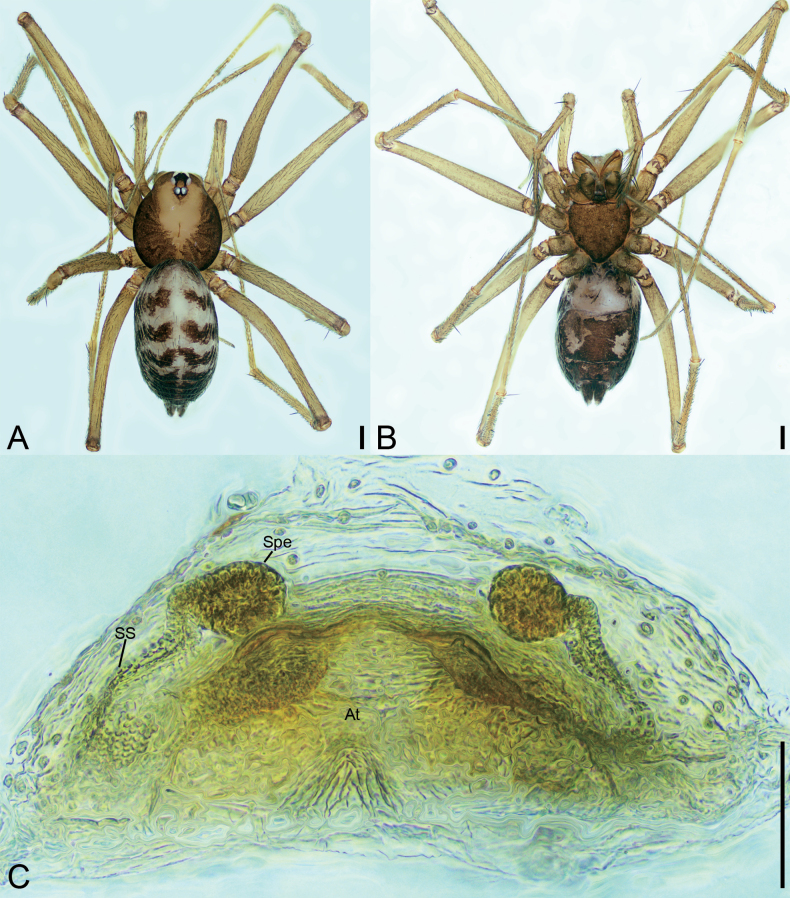
*Longileptonetarenzhouensis* sp. nov., female paratype **A** habitus, dorsal view **B** same, ventral view **C** vulva, dorsal view. Abbreviations: At – atrium, Spe – spermathecae, SS – spermathecae stalk. Scale bars: 0.2 mm (**A, B**); 0.1 mm (**C**).

Figs 19 , 20 , 21 , 28E Vernacular name: 仁洲长弱蛛

##### Material examined.

***Holotype***: ♂, **China**: Fujian Province, Fuzhou City, Minhou County, Jingxi Town, Renzhou Village, Sandiejing Forest Park, 26°16'3.31"N, 119°09'5.08"E, 24.X.2023, Y. Yao, J. Gong, R. Zhao & M. Wu leg. (Lep-14). ***Paratype***: 2 ♂, 2 ♀, the same data as the holotype (Lep-14); 1 ♂, 29.X.2023, other data as same as the holotype (Lep-14).

##### Diagnosis.

The male of this species can be easily distinguished from other members of this genus by the very long curved spine-like prolateral sclerite with a feathery tip (Figs 19C–E, 20). The female resembles *L.zhuxian* Wang & Li, 2020 (Wang et al. 2020: 700, fig. 16C) in having spheroid spermathecae and the subtrapezoid atrium, but can be separated by the slightly curved spermathecal stalk (vs waved) (Fig. 21C).

##### Description.

**Male** (holotype). Habitus as in Fig. 19A, B. Total length 2.73. Carapace 1 long, 0.9 wide. Eye sizes and interdistances: ALE 0.08, PME 0.08, PLE 0.08; ALE–PME 0.13, PLE–PLE 0.12, PLE–PME 0.04; AER 0.16, PER 0.20. Clypeus 0.22 high. Chelicerae (Fig. 19B) with nine promarginal and ten retromarginal teeth. Endites with several long setae laterally. Labium sub-rectangular, anteriorly with more than three pairs of strong setae. Sternum (Fig. 19B) shield-shaped, slightly longer than wide, with sparse setae on surface, posterior end blunt. Leg measurements: I 8.38 (2.21, 0.33, 2.62, 2.19 1.03); II 5.62 (1.57, 0.31, 1.86, 1.26, 0.62); III 5.21 (1.37, 0.34, 1.40, 1.26, 0.84); IV 6.95 (1.76, 0.33, 2.22, 1.68, 0.96). Pedicel 0.11. Abdomen 1.60 long, 1.01 wide.

***Coloration*** (Fig. 19A, B). Carapace yellow, with radial yellow stripes submarginally, clypeus mottled. Chelicerae and endites yellow to dark brown, mottled. Labium brown, mottled. Sternum dark brown, mottled. Legs yellow to dark brown, mottled. Abdomen dorsally yellowish, with three pairs of black spots, anterior part mottled; venter black, mottled.

***Palp*** (Figs 19C–E, 20). Femur with three rows of short strong spines ventrally, dorsally, and prolaterally; patella lacking spine; tibia with one columnar apophysis, armed with one straight spine, and one long and very thick spine, slightly shorter than the apophysis. Cymbium with a distinct constriction medially. Tip of bulb: one broad median apophysis, with a furcate tip, including one long membranous and one hook-shaped; prolateral lobe finger-like; conductor membranous, touching with prolateral sclerite; prolateral sclerite waved, needle-like, with a feathery tip. Embolus short hook-shaped, with a broad base.

**Female** (paratype). Habitus as in Fig. 21A, B. Total length 2.23. Carapace 0.94 long, 0.86 wide. Eye sizes and interdistances: ALE 0.09, PME 0.08, PLE 0.08; ALE–PME 0.11, PLE–PLE 0.11, PLE–PME 0.04; AER 0.17, PER 0.18. Clypeus 0.19 high. Chelicerae (Fig. 21B) with nine promarginal and ten retromarginal teeth. Endites with several long spines anterolaterally. Sternum (Fig. 21B) shield-shaped, nearly as long as wide, with dense scale-like surface, lateral margin thickened, posterior end blunt. Leg measurements: I 7.40 (1.98, 0.31, 2.42, 1.57, 1.12); II 5.56 (1.51, 0.33, 1.63, 1.23, 0.86); III 4.59 (1.33, 0.31, 1.21, 1.03, 0.71); IV 6.70 (1.98, 0.33, 1.96, 1.47, 0.96). Pedicel 0.04. Abdomen 1.41 long, 0.84 wide.

***Vulva*** (Fig. 21C). Internal genitalia with sub-trapezoidal atrium, spherical spermathecae, and slightly curved spermathecal stalk.

##### Distribution.

Known only from the type locality in Fujian Province, China (Fig. 29).

##### Etymology.

The name is taken from the type locality.

#### ﻿ Longileptoneta tianmenensis

Taxon classificationAnimaliaAraneaeLeptonetidae

Yao & Liu sp. nov.

9C94CE91-4B1A-537B-BF84-C9A1520E5DF4

https://zoobank.org/DC584FE1-B618-452A-96C1-97625ED931B0

**Figure 22.**
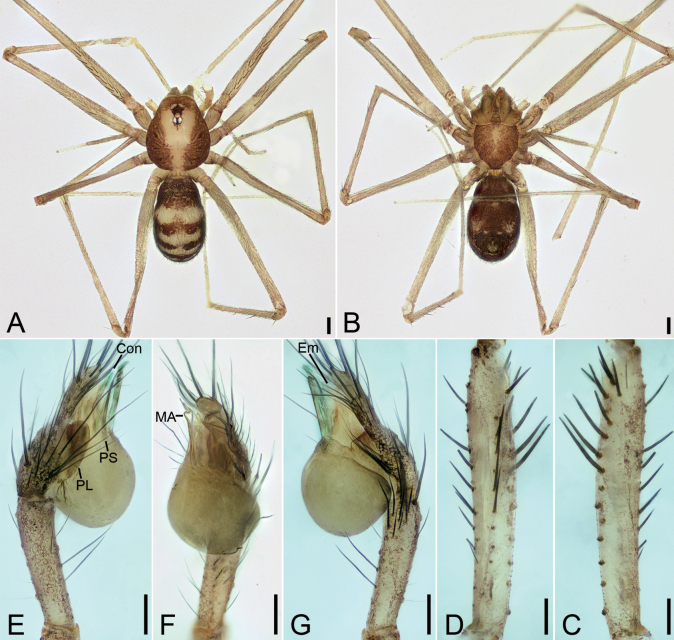
*Longileptonetatianmenensis* sp. nov., male holotype **A** habitus, dorsal view **B** same, ventral view **C** femur, retrolateral view **D** same, prolateral view **E** palp, prolateral view **F** same, ventral view **G** same, retrolateral view. Abbreviations: Con – conductor, Em – embolus, MA – medial apophysis, PL – prolateral lobe, PS – prolateral sclerite. Scale bars: 0.2 mm (**A, B**); 0.1 mm (**C–G**).

**Figure 23.**
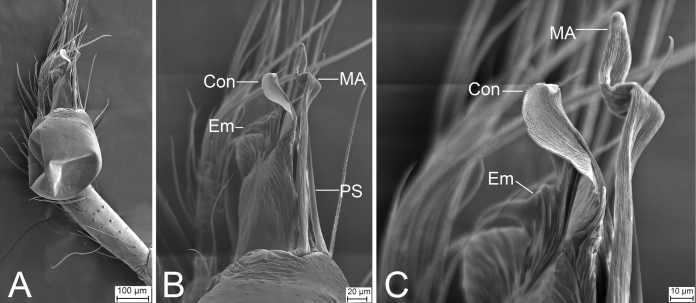
SEM micrographs of *Longileptonetatianmenensis* sp. nov., male right palp, holotype **A** ventral view **B** detail of tegular apophysis, ventral view **C** same, ventral view. Abbreviations: Con – conductor, Em – embolus, MA – medial apophysis, PS – prolateral sclerite.

**Figure 24.**
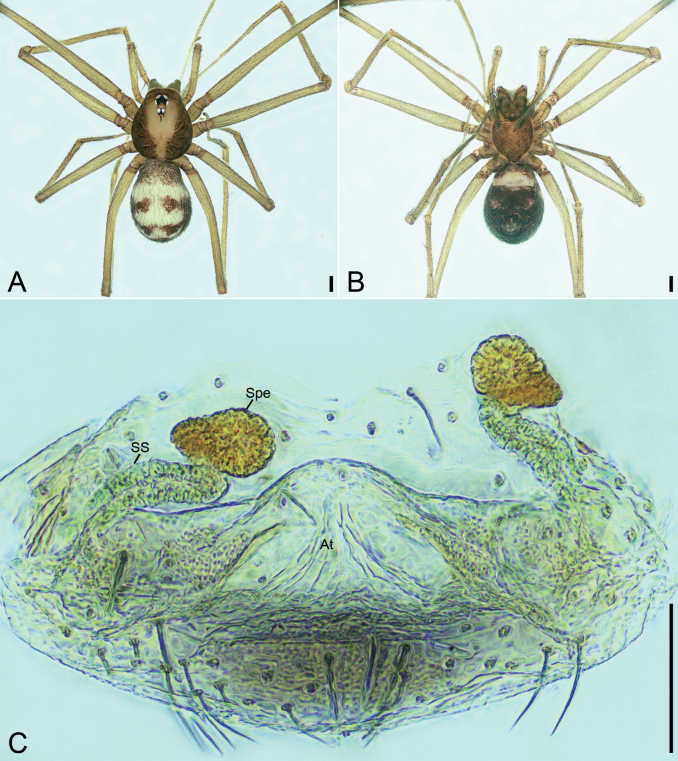
*Longileptonetatianmenensis* sp. nov., female paratype **A** habitus, dorsal view **B** same, ventral view **C** vulva, dorsal view. Abbreviations: At – atrium, Spe – spermathecae, SS – spermathecae stalk. Scale bars: 0.2 mm (**A, B**); 0.1 mm (**C**).

Figs 22 , 23 , 24 , 28F Vernacular name: 天门山长弱蛛

##### Material examined.

***Holotype***: ♂, **China**: Fujian Province, Fuzhou City, Yongtai County, Geling Town, Yangxi Village, Tianmen Mountain, 25°49'5.34"N, 119°00'40.79"E, 10.IV.2023, J. Gong, R. Zhao & M. Wu leg. (Lep-11). ***Paratype***: 2 ♀, 14.X.2023, Y. Yao & M. Wu leg, other data as same as the holotype (Lep-11).

##### Diagnosis.

The male of this species is similar to that of *L.huboliao* sp. nov. in having the femur with three rows of strong spines, one columnar tibial apophysis, armed with a short spine-like tip on palp and a needle-like prolateral sclerite, but can be easily distinguished from it by the rod-like median apophysis (vs lacking) the membranous conductor lacking a serrulate tip (vs present) (Figs 22C–G, 23). The female resembles *L.huboliao* sp. nov. in having a bell-like atrium and the spermathecal stalk lacking a spiral twist, but can be separated by the oval spermathecae (vs tube-shaped) (Fig. 24C).

##### Description.

**Male** (holotype). Habitus as in Fig. 22A, B. Total length 2.14. Carapace 0.94 long, 0.82 wide. Eye sizes and interdistances: ALE 0.10, PME 0.08, PLE 0.07; ALE–PME 0.10, PLE–PLE 0.13, PLE–PME 0.03; AER 0.17, PER 0.18. Clypeus 0.18 high. Chelicerae (Fig. 22B) with nine promarginal and eight retromarginal teeth. Endites with several long setae laterally. Labium sub-rectangular, anteriorly with more than two pairs of strong setae. Sternum (Fig. 22B) shield-shaped, slightly longer than wide, with sparse setae on surface, posterior end blunt. Leg measurements: I 8.41 (2.63, 0.23, 2.94, 2.06, 0.55); II 6.62 (1.88, 0.24, 1.99, 1.44, 1.07); III 5.34 (1.50, 0.24, 1.50, 1.27, 0.83); IV 7.05 (1.90, 0.29, 2.12, 1.93, 0.81). Pedicel 0.06. Abdomen 1.11 long, 0.70 wide.

***Coloration*** (Fig. 22A, B). Carapace yellowish to black-brown, with dark radial stripes on lateral margin. Chelicerae yellow to brown, mottled. Labium dark brown. Sternum yellow to black, mottled. Legs yellow, mottled. Abdomen, dorsally yellowish, with two pairs of spots; venter dark.

***Palp*** (Figs 22C–G, 23). Femur with three rows of short strong spines ventrally, dorsally, and prolaterally; patella lacking strong spine; tibia with a columnar apophysis, armed with one short straight spine. Cymbium with a distinct constriction medially. Tip of bulb: one rod-like median apophysis, thick; prolateral lobe oval, lamellar; conductor membranous, shorter than median apophysis. Embolus hook-shaped, beneath conductor.

**Female** (paratype). Habitus as in Fig. 24A, B. Total length 2.22. Carapace 1.01 long, 0.88 wide. Eye sizes and interdistances: ALE 0.09, PME 0.07, PLE 0.06; ALE–PME 0.12, PLE–PLE 0.12, PLE–PME 0.06; AER 0.17, PER 0.19. Clypeus 0.11 high. Chelicerae (Fig. 24B) with nine promarginal and twelve retromarginal teeth. Endites with several long spines anterolaterally. Sternum (Fig. 24B) shield-shaped, nearly as long as wide, with dense scale-like surface, lateral margin thickened, posterior end blunt. Leg measurements: I 9.06 (2.42, 0.32, 2.86, 2.21, 1.25); II 6.69 (1.73, 0.33, 2.07, 1.65, 0.91); III 5.20 (1.46, 0.31, 1.39, 1.20, 0.84); IV 6.65 (2.02, 0.27, 1.96, 1.48, 0.92). Abdomen 1.2 long, 0.88 wide.

***Vulva*** (Fig. 24C). Internal genitalia with sub-trapezoidal atrium, slightly swollen spermathecae, and convoluted spermathecal stalk including three coils.

##### Distribution.

Known only from the type locality in Fujian Province, China (Fig. 29).

##### Etymology.

The name is taken from the type locality.

### ﻿Genus *Pararana* Lin & Li, 2022

#### ﻿ Pararana mingxuani

Taxon classificationAnimaliaAraneaeLeptonetidae

Yao & Liu sp. nov.

28041C1D-AC15-59AB-9E4C-9E69A048484A

https://zoobank.org/CDB6306E-1FF8-49B4-B68C-F2ECEA5FD3D5

**Figure 25.**
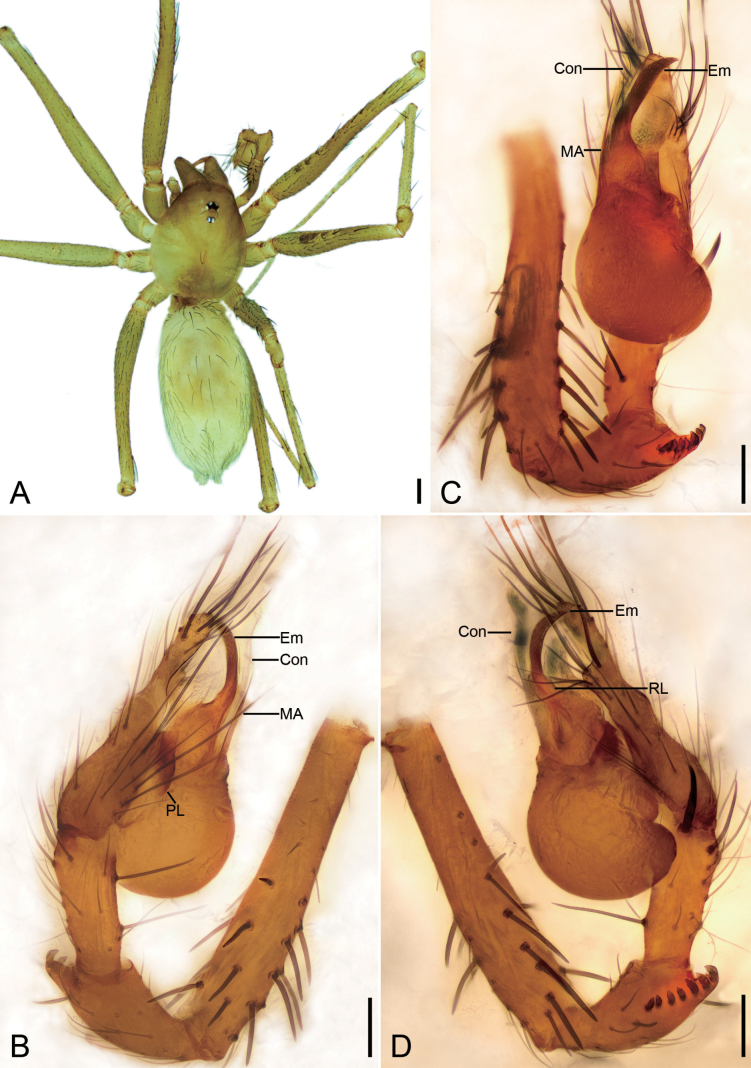
*Pararanamingxuani* sp. nov., male holotype **A** habitus, dorsal view **B** palp, prolateral view **C** same, ventral view **D** same, retrolateral view. Abbreviations: Con – conductor, Em – embolus, MA – medial apophysis, PL – prolateral lobe, RL – retrolateral lobe. Scale bars: 0.2 mm (**A**); 0.1 mm (**B–D**).

**Figure 26.**
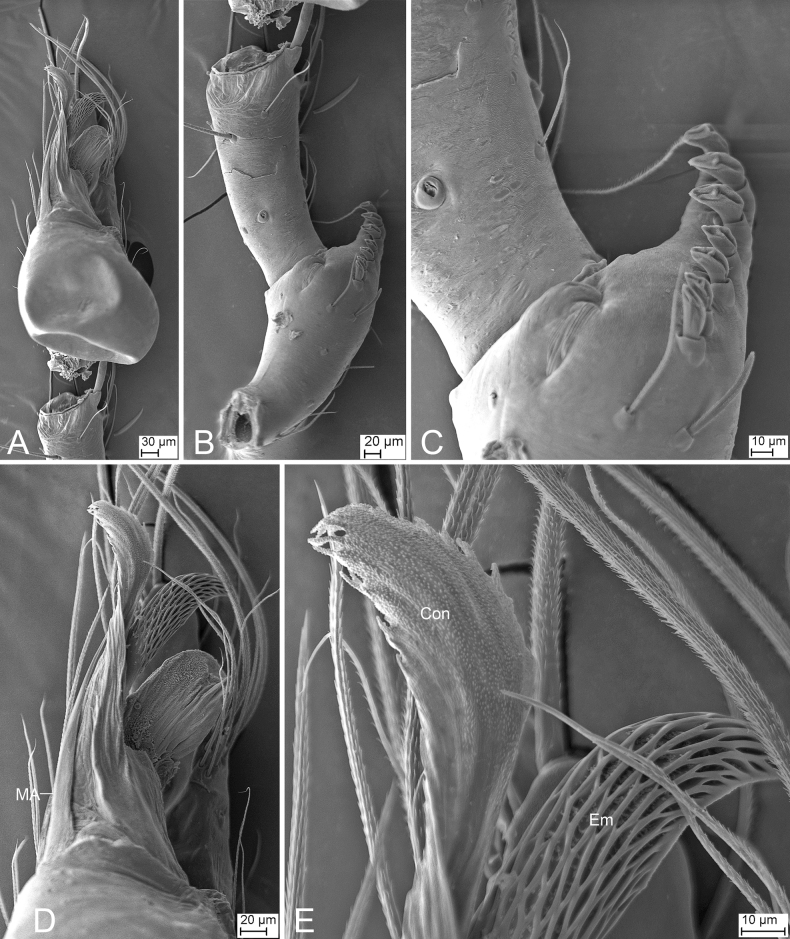
SEM micrographs of *Pararanamingxuani* sp. nov., male palp **A** ventral view **B** patella, retrolateral view **C** detail of patellar spines, retrolateral view **D** detail of tegular apophysis, ventral view **E** detail of conductor and embolus, ventral view. Abbreviations: Con – conductor, Em – embolus, MA – medial apophysis.

**Figure 27.**
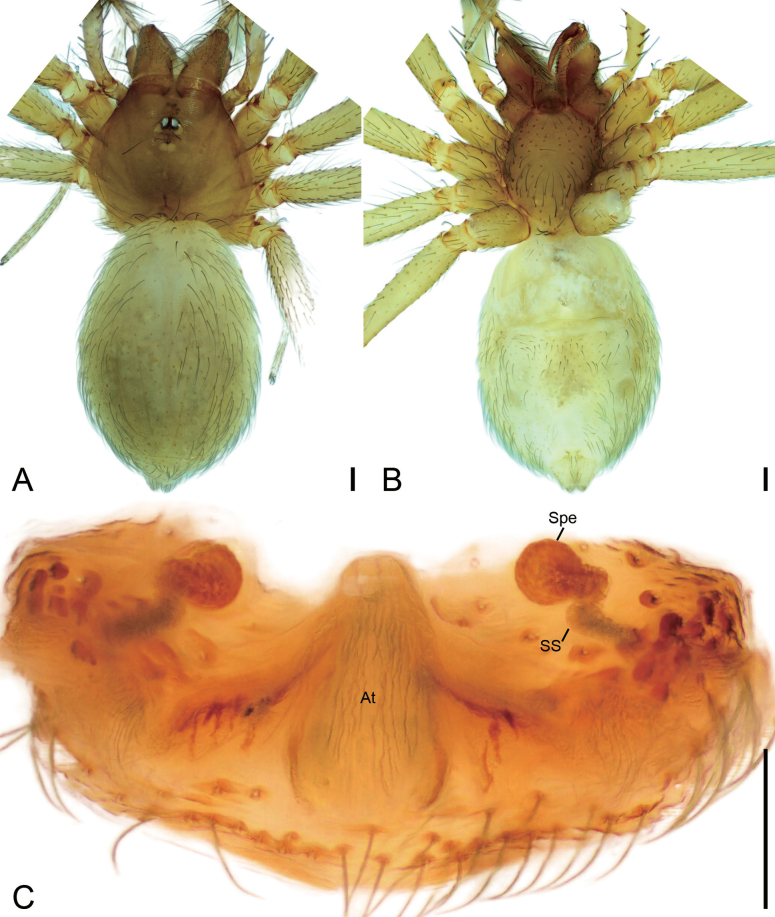
*Pararanamingxuani* sp. nov., female paratype **A** habitus, dorsal view **B** same, ventral view **C** vulva, dorsal view. Abbreviations: At – atrium, Spe – spermathecae, SS – spermathecae stalk. Scale bars: 0.1 mm.

**Figure 28.**
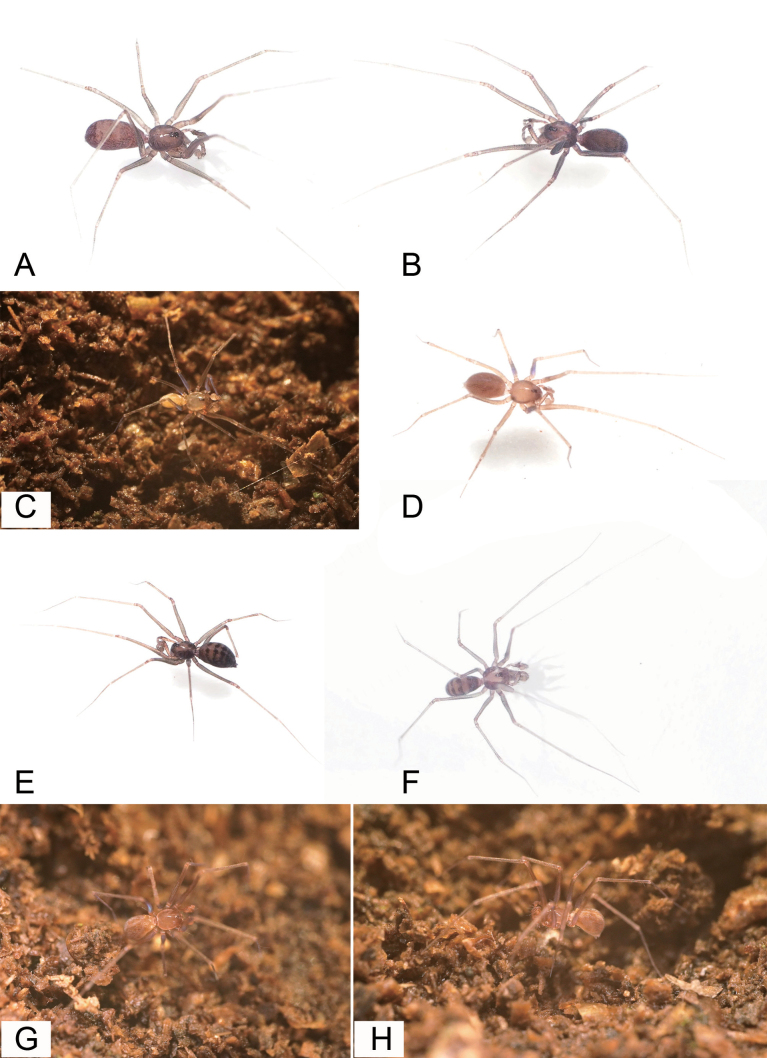
Photographs of living specimens from China. **A***Longileptonetaguadunensis* sp. nov., **B***L.huboliao* sp. nov. **C***L.jiaxiani* sp. nov. **D***L.letuensis* sp. nov. **E***L.renzhouensis* sp. nov. **F***L.tianmenensis* sp. nov. **G, H***Pararanamingxuani* sp. nov.

Figs 25 , 26 , 27 , 28G, H Vernacular name: 明轩拟正弱蛛

##### Material examined.

***Holotype***: ♂, Fujian Province, Fuzhou City, Yongtai County, Geling Town, Xiyang Village, Tianmen Mountain, 25°49'7.6"N, 119°1'5.07"E, 10.IV.2023, R. Zhao, J. Gong & M. Wu leg. (Lep-9). ***Paratype***: 1 ♂, 1 ♀, Fujian Province, Fuzhou City, Minhou County, Nanyu Town, 25°58'24.05"N, 119°13'15.87"E, 5.VI.2023, Y. Yao, W. Zhang, M. Wu & R. Zhao leg. (Lep-9).

##### Diagnosis.

The male of this species is similar to that of *Pararanagaofani* Lin & Li, 2022 (Lin et al. 2022: 217, figs 17A–C, 18A, B) in having the cymbium with a notch and the swollen patella, but can be easily separated by the patella with seven short tooth-like spines (vs four long relatively thick spines), the tibia with a thick spine (vs absent), the long lamellar median apophysis (vs the relatively short horn-like median apophysis) and the slightly curved rod-like embolus (vs horn-like) (Figs 25B–D, 26). The female can be easily distinguished by the oval atrium and the short S-shaped spermathecal stalk (Fig. 27C).

##### Description.

**Male** (holotype). Habitus as in Fig. 25A. Total length 2.32. Carapace 0.97 long, 0.81 wide. Eye sizes and interdistances: ALE 0.05, PME 0.04, PLE 0.05; ALE–PME 0.10, PLE–PLE 0.06, PLE–PME 0.05; AER 0.11, PER 0.13. Clypeus 0.23 high. Chelicerae (Fig. 25A) with 13 promarginal and five retromarginal teeth. Endites with several long setae laterally and seven leaf-shaped setae anteriorly. Labium sub-rectangular, anterolaterally with two pairs of strong setae and anteriorly with eight setae. Sternum shield-shaped, longer than wide, with sparse setae on surface, posterior end very blunt. Leg measurements: I (2.00, 0.33, other segments broken); II 5.86 (1.54, 0.32, 1.78, 1.42, 0.80); III 4.78 (1.35, 0.29, 1.15, 1.19, 0.80); IV (1.77, 0.28, other segments broken). Pedicel 0.08. Abdomen 1.36 long, 0.76 wide.

***Coloration*** (Fig. 25A). Carapace yellow, with dark radial stripes. Chelicerae yellow to brown. Endites yellow to dark brown. Labium yellow brown. Legs with dark annulations on each segment except tarsi. Abdomen yellow.

***Palp*** (Figs 25B–D, 26). Femur with four rows of short strong spines ventrally, dorsally, and prolaterally; patella expanded, with seven stout spines; tibia with a very thick spine retrolaterally. Cymbium with a notch subapically. Bulb: prolateral lobe banded, long; embolus rod-like, slightly curved, with a broad base and a net-shaped surface; median apophysis lamellar, shorter than conductor; conductor membranous, with serrulate margin; retrolateral lobe blunt, tongue-shaped, touching base of conductor.

**Female** (paratype). Habitus as in Fig. 27A, B. Total length 1.83. Carapace 0.86 long, 0.76 wide. Eye sizes and interdistances: ALE 0.06, PME 0.04, PLE 0.05; ALE-PME 0.07, PLE-PLE 0.07, PLE-PME 0.05, AER 0.09, PER 0.14, Clypeus 0.20 high. Chelicerae (Fig. 27B) with 12 promarginal and four retromarginal teeth. Endites with several long spines anterolaterally. Sternum (Fig. 27B) shield-shaped, nearly as long as wide, with dense scale-like surface, lateral margin thickened, posterior end blunt. Leg measurements: I 4.68 (1.20, 0.24, 1.40, 1.13, 0.71); II 4.74 (1.18, 0.20, 1.49, 1.17, 0.70); III 3.03 (0.82, 0.20, 0.79, 0.74, 0.48); IV (1.03, 0.21, other segments broken). Pedicel 0.04. Abdomen 1.17 long, 0.84 wide.

***Coloration*** (Fig. 27A, B). Darker than male.

***Vulva*** (Fig. 27C). Internal genitalia with bell-shaped atrium, the spheroidal spermathecae and the S-shaped spermathecal stalk including two turns.

##### Distribution.

**Figure 29.**
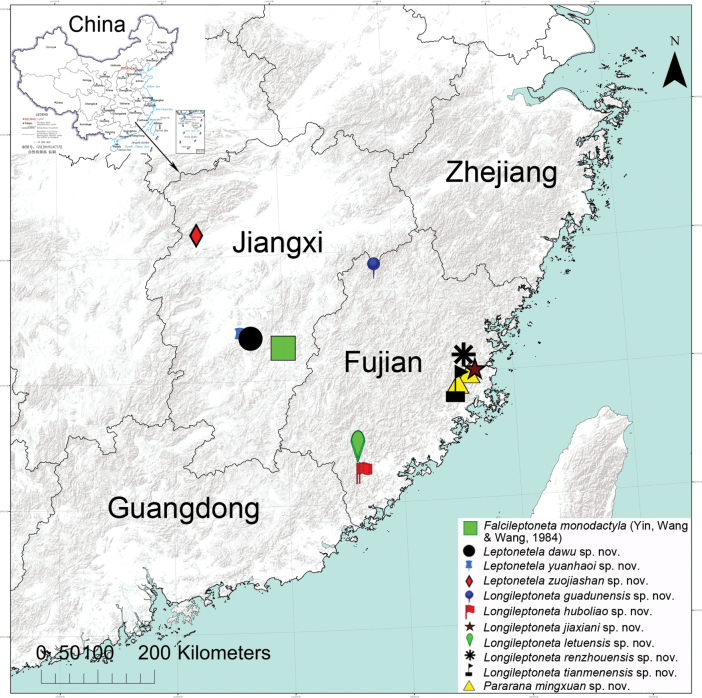
Records of *Falcileptonetamonodactyla* (Yin, Wang & Wang, 1984), *Leptoneteladawu* sp. nov., *L.yuanhaoi* sp. nov. and *L.zuojiashan* sp. nov. from Jiangxi; *Longileptonetaguadunensis* sp. nov., *L.huboliao* sp. nov., *L.jiaxiani* sp. nov., *L.letuensis* sp. nov., *L.renzhouensis* sp. nov., *L.tianmenensis* sp. nov., and *Pararanamingxuani* sp. nov. from Fujian, China.

Known only from the type locality in Fujian Province, China (Fig. 29).

##### Etymology.

The species is named after Mr Mingxuan Wu, who collected the type specimens.

## ﻿Discussion

At present, China is the most diverse region for Leptonetidae (144 species), followed by the USA (60 species), Korea (58 species), Japan (53 species), France (26), and Greece (16), with few species found in the remaining regions (29 species; WSC 2023). Within 15 years (2008 to 2022) the total number of leptonetid species recorded from China increased six times (WSC 2023), mostly after the profusely illustrated revisions of the Chinese representatives by Wang et al. (2017) and the Japanese species by Ballarin and Eguchi (2022). These two studies have revealed remarkable sexually dimorphic traits and relevant morphological features that have provided useful information for the present taxonomic work.

It is interesting to note that the species *Falcileptonetamonodactyla* has no also been found from Jiangxi Province. Considering the locality of the holotype, Yanling county in Hunan province, it is likely that this species is more widely distributed in the Hunan and Jiangxi provinces.

The *Longileptoneta* species are very difficult to differentiate as their embolus is hidden in the tegular apophyses. Although the genera *Falcileptoneta* and *Longileptoneta* are clearly distinguished from all other leptonetid genera, ambiguity can occur in cave species lacking eyes, such as *Falcileptonetataizhensis* (Chen & Zhang, 1993), *Longileptonetagutan* Wang & Li, 2020, and *L.shenxian* (Wang et al. 2020). A very obvious feature reveals that they are living in caves and become vestigial. Each of these two genera seems to be monophyletic, which need to be confirmed by future phylogenetic studies.

The genus *Pararana* Lin & Li, 2022 was monotypic before this work and described based on a single male specimen (Lin et al. 2022). The diagnostic characters of the genus are inadequate as no females are known for the type species *Pararanagaofani* Lin & Li, 2022 (Lin et al. 2022). Based on the female of *P.mingxuani* sp. nov., this genus can be characterized by a long atrium and very short spermathecal stalks. Since more *Pararana* species from China can be expected to be discovered, this genus will be more easily understood in future research.

## Supplementary Material

XML Treatment for Falcileptoneta monodactyla

XML Treatment for Leptonetela dawu

XML Treatment for Leptonetela yuanhaoi

XML Treatment for Leptonetela zuojiashanensis

XML Treatment for Longileptoneta guadunensis

XML Treatment for Longileptoneta huboliao

XML Treatment for Longileptoneta jiaxiani

XML Treatment for Longileptoneta letuensis

XML Treatment for Longileptoneta renzhouensis

XML Treatment for Longileptoneta tianmenensis

XML Treatment for Pararana mingxuani

## ﻿Additional information

### Author ORCIDs

Bin-Lu Liu https://orcid.org/0009-0009-3080-8987

Yan-bin Yao https://orcid.org/0000-0002-2560-9299

Zi-Min Jiang https://orcid.org/0000-0001-9044-3312

Yong-hong Xiao https://orcid.org/0000-0002-8074-9366

Ke-Ke Liu https://orcid.org/0000-0001-7822-3667

### Data availability

All of the data that support the findings of this study are available in the main text.
